# Evaluation of clinical outcomes in patients treated with heparin or direct thrombin inhibitors during extracorporeal membrane oxygenation: a systematic review and meta-analysis

**DOI:** 10.1186/s12959-022-00401-2

**Published:** 2022-07-28

**Authors:** René M’Pembele, Sebastian Roth, Aljoscha Metzger, Anthony Nucaro, Alexandra Stroda, Amin Polzin, Markus W. Hollmann, Giovanna Lurati Buse, Ragnar Huhn

**Affiliations:** 1 Department of Anesthesiology, Medical Faculty and University Hospital Duesseldorf, Heinrich-Heine-University, Duesseldorf, Germany; 2 Department of Cardiology, Pulmonology and Vascular Medicine, Medical Faculty and University Hospital Duesseldorf, Heinrich-Heine-University, Duesseldorf, Germany; 3 Department of Anesthesiology, Amsterdam University Medical Center (AUMC), Location AMC, Amsterdam, The Netherlands; 4 Department of Anesthesiology, Kerckhoff Heart and Lung Center, Bad Nauheim, Germany

**Keywords:** Bivalirudin, Argatroban, Anticoagulation, Bleeding, Thrombosis, Mechanical circulatory support

## Abstract

**Background:**

The number of patients treated with extracorporeal membrane oxygenation (ECMO) devices is increasing. Anticoagulation therapy is crucial to prevent thrombosis during ECMO therapy. Predominantly, heparin has been used as primary anticoagulant but direct thrombin inhibitors (DTI) have been established as alternatives. The aim of this systematic review and meta-analysis was to evaluate clinical outcomes in patients treated with heparin compared to different DTI during ECMO.

**Methods:**

A systematic search was conducted. Full scientific articles were sought for inclusion if heparin anticoagulation was compared to DTI (argatroban/bivalirudin) in ECMO patients. Risk of bias was assessed by Newcastle Ottawa scale. Primary endpoint was in-hospital mortality. Bleeding events, thrombotic events, hours of ECMO support, days of hospital stay, percentage of time within therapeutic range and time to therapeutic range were extracted from full texts as secondary endpoints. Results were presented as Forrest-plots. GRADE was used for confidence assessment in outcomes.

**Results:**

Systematic search identified 4.385 records, thereof 18 retrospective studies for a total of 1942 patients, complied with the predefined eligibility criteria:15 studies investigated bivalirudin and 3 studies investigated argatroban versus heparin. Risk of bias was high for most studies. In-hospital mortality, major bleeding events and pump-related thrombosis were less frequent in DTI group as compared to heparin [mortality—OR 0.69, 95% CI 0.54–0.86; major bleeding—OR 0.48, 95% CI 0.29–0.81; pump thrombosis—OR 0.55, 95% CI 0.40–0.76]. Additionally, percentage of time within therapeutic range was higher for DTI [SMD 0.54, 95% CI 0.14–0.94]. GRADE approach revealed a very low level of certainty for each outcome.

**Conclusion:**

In this meta-analysis, DTI and especially bivalirudin showed beneficial effects on clinical outcomes in ECMO patients as compared to heparin**.** However, due to the lack of randomized trials, certainty of evidence is low.

**Trial Registration:**

This systematic review and meta-analysis was prospectively registered at PROSPERO data base (reference number CRD42021237252).

**Graphical Abstract:**

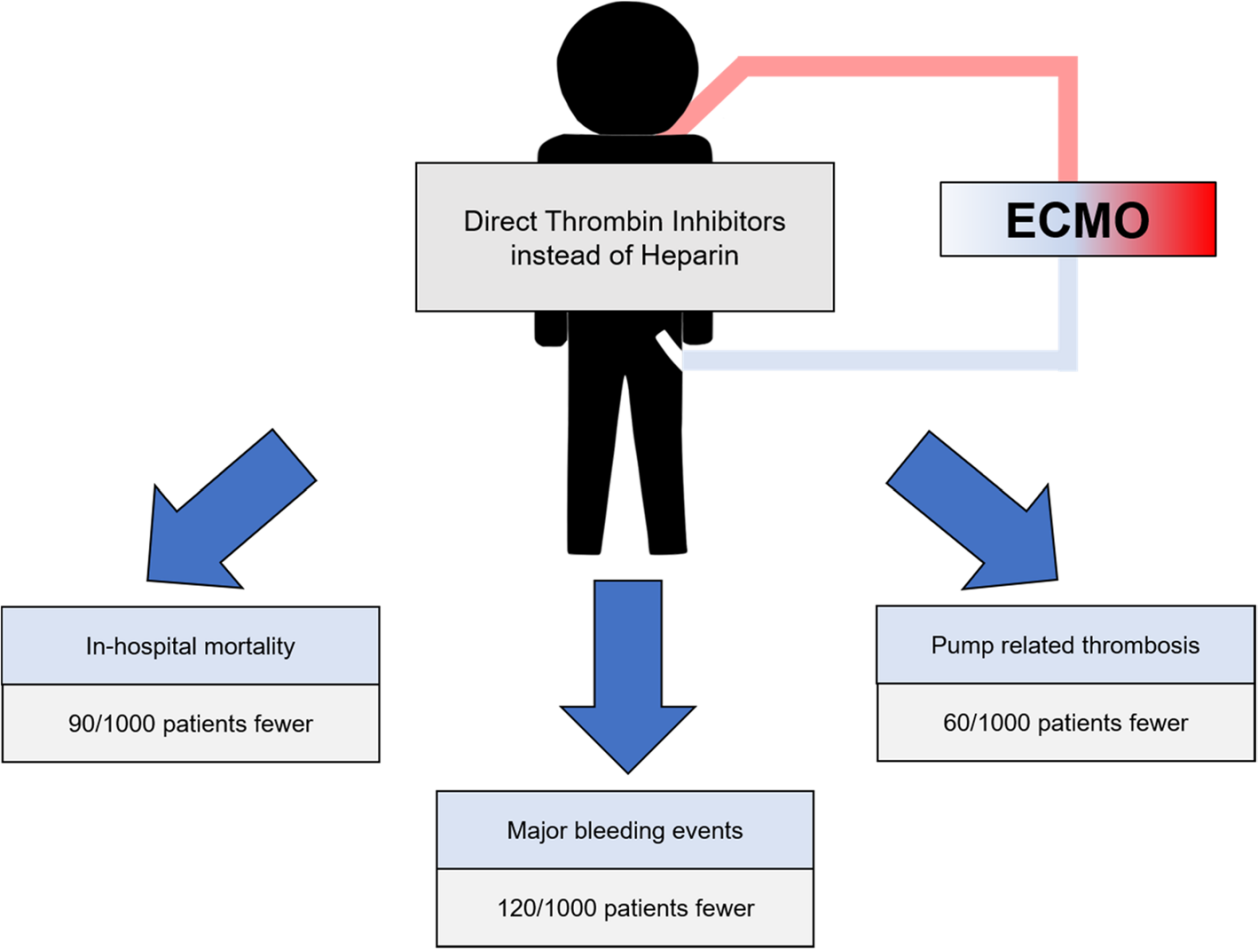

**Supplementary Information:**

The online version contains supplementary material available at 10.1186/s12959-022-00401-2.

## Introduction

Numbers of patients treated with extracorporeal membrane oxygenation (ECMO) devices have been constantly increasing during the past decade [1]. Frequent indications for ECMO therapy are cardiogenic shock (CS), respiratory failure, severe sepsis, or failure to wean from cardiopulmonary bypass after cardiac surgery [2, 3]. During extracorporeal circulation the exposure to exogenous surfaces leads to activation of blood coagulation [4]. Therefore, anticoagulation therapy is mandatory to prevent thrombosis during ECMO therapy. Heparin is used in most centers for anticoagulation in ECMO patients [5, 6]. However, heparin induced thrombocytopenia and heparin resistance are conditions frequently requiring the use of alternative anticoagulants [7, 8]. In this context direct thrombin inhibitors (DTI) like bivalirudin and argatroban have been established as alternatives [8]. Previous research indicate that titration of anticoagulation within therapeutic range might be more feasible with DTI as compared to heparin [9, 10]. Maintenance of therapeutic anticoagulation is crucial, as subtherapeutic doses may results in thrombotic and supratherapeutic doses in bleeding complications with deleterious impact on outcome of ECMO patients. Therefore, some centers primarily use DTI for anticoagulation during ECMO as they might have beneficial influence on outcome [11]. A meta-analysis recently indicated a survival benefit and a reduced incidence of thrombosis in adults treated with bivalirudin as compared to heparin during ECMO therapy [12, 13]. For argatroban, while systematic reviews were conducted, meta-analyses are lacking [14]. Especially comparison of evidence between different DTIs versus heparin has not been demonstrated. The aim of this systematic review and meta-analysis was to evaluate clinical outcomes (in hospital mortality, bleeding complications, thrombotic complications, length of hospital stay, and ECMO duration) in patients treated with Heparin compared to DTI during ECMO and to compare evidence for different DTI by subgroup analysis.

## Methods

The report of this systematic review and meta-analysis follows the Preferred Reporting Items for Systematic Reviews and Meta-Analyses (PRISMA) guidelines. The protocol and predefined analysis plan is attached as Supplementary material (Supplement 1). The review was registered at PROSPERO on 22th March 2021 (CRD42021237252).

### PICO-statement

Population of interest were adult and pediatric patients treated with venoarterial or venovenous ECMO. Intervention was DTI (bivalirudin or argatroban) as primary anticoagulation strategy during ECMO. Anticoagulation using heparin during ECMO was the control strategy. Primary endpoint was in-hospital mortality. Secondary outcomes were number of patients with major and minor bleeding events, patient- and device-related thrombotic or ischemic events during ECMO run, hours of ECMO support, length of hospital stay in days, percentage of activated partial thromboplastin time (aPTT) within therapeutic window and hours to therapeutic aPTT levels.

### Eligibility criteria

Published and unpublished randomized controlled trials, prospective or retrospective cohort studies and case–control studies investigating DTI versus heparin in ECMO patients were eligible. Study selection was restricted to English language and only full scientific reports were included. Poster presentations, conference abstracts, systematic reviews and meta-analysis, studies not comparing DTI to heparin in ECMO patients, studies in which patients received DTI only as secondary anticoagulation strategy and studies not reporting on any of the endpoints mentioned above were excluded.

### Information sources & search strategy

The following medical libraries were searched for eligible studies published from inception to January 2022: Pubmed/Medline, Cochrane library, CINAHL, Embase. Medical subject headings (MeSh), field terms, text words and Boolean operators were combined in a block building search. Search term contained “extracorporeal membrane oxygenation”, “bivalirudin”, “argatroban”, “direct thrombin inhibitor”, “heparin”, “anticoagulation”, “embolism and thrombosis”, “hemorrhage”, “survival” and “adverse drug event” amongst others. First date of search was 18^th^ August 2021, last date of search was 20^th^ January 2022. Detailed search strategies are listed in supplement 2. Additionally, the local medical library of the University of Duesseldorf (ULB) was searched and authors of eligible studies were contacted for unpublished data.

### Selection process

Two independent researchers screened titles and abstracts of search results from each medical library and retrieved eligible studies. In the second step, the two researchers independently selected studies fulfilling the predefined eligibility criteria based on the full text. After each step, disagreements between both researchers were discussed. No automation tools were used in this process.

### Data collection & data items

Data regarding study characteristics and endpoints was extracted from full text, tables and supplements by one reviewer. Entries were independently checked by a second investigator.

If data items (primary or secondary outcomes) were not extractable from publications, authors were contacted via email and requested to complement missing data. Additionally, authors were asked to check the extracted data from their studies in the final version of this manuscript. In case outcomes were available before and after adjustment (for example propensity score matching), we included adjusted data into analysis. If data was not available in desired measurement unit authors were contacted to provide this data. Apart from primary and secondary outcomes, other variables were sought as study characteristics: Study design, number of patients, type of anticoagulation, sex, mean age, type of ECMO, indication for ECMO, aPTT-aim and regime for dosage of anticoagulation. Again, authors were contacted for missing information.

### Study risk of bias assessment

Risk of bias was examined separately by two independent investigators using the Newcastle–Ottawa-Scale for non-randomized trials [15]. Study quality was determined as good, fair or poor quality according to scale ratings. Good quality was defined as 3–4 points within selection section and 1–2 points within comparability section and 2–3 points within outcome section. Fair quality was defined as 2 points within selection section and 1–2 points within comparability section and 2–3 points within outcome section. Poor quality was defined as 0–1 points within selection section or 0 points within comparability section or 0–1 point within outcome section.

### Effect measures for outcomes

For all dichotomous outcomes Odds ratio (OR) was used as effect measure for data synthesis and presentation of results. Results for continuous outcomes were presented as standardized mean difference (SMD).

### Methods of data synthesis and statistical analysis

Meta-analysis was performed for primary and secondary outcomes. Study data were included into analysis if the study reported separately outcomes for heparin and DTI patients. No data conversion was conducted. Study results were presented as tables. Additionally, Forrest plots with pooled estimates of effect were generated for each outcome. Assuming that effects differed across studies a random-effects model was used to account for within and between study variance. To assess for statistical heterogeneity between studies, I^2^ tests and Cochrane-Q tests were conducted. Subgroup analysis for adult versus pediatric patients, risk of bias and argatroban versus bivalirudin were conducted to explore possible reasons for heterogeneity. These subgroups were defined a priori. Planned sensitivity analysis was performed for analysis methods by using fixed effects models instead of random effects models and using risk ratio (RR) and risk difference instead of OR for dichotomous outcomes. For continuous variables, MD for individual scale measures were explored and compared to SMD.

Funnel plots were created for each outcome to address for reporting bias. For statistical analysis Review Manager (RevMan) [Computer program]. Version 5.4. (The Cochrane Collaboration, 2020) was used and a *p*-value of < 0.05 was considered as significant, refuting the null hypothesis. Level of confidence for each outcome was assessed by GRADE approach and presented as summary of findings table.

## Results

### Study selection

**Fig. 1:**
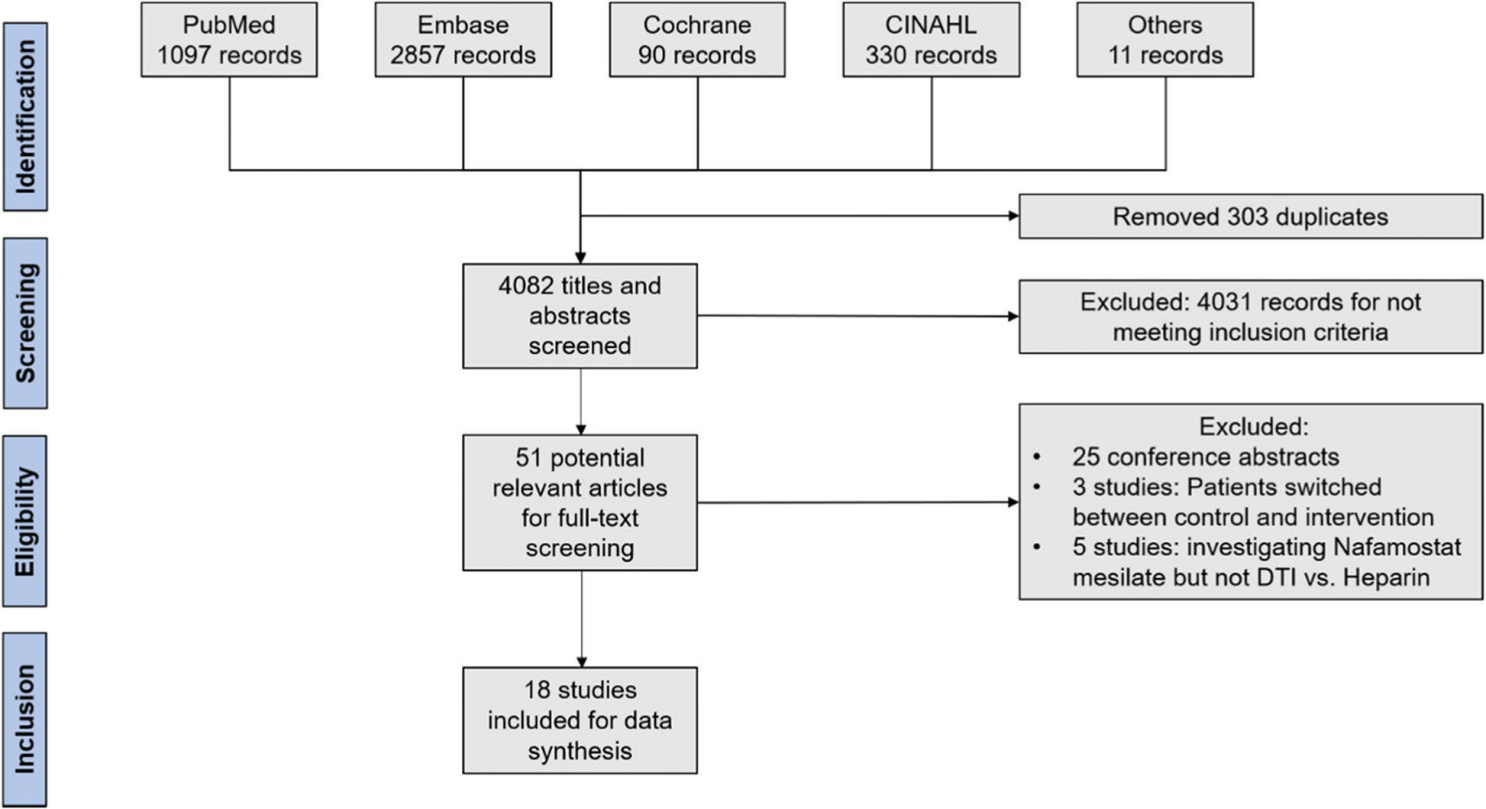
Flow-chart of study selection process

The systematic search identified a total of 4.385 records. After removing of 303 duplicates 4.082 records remained for screening of titles and abstracts. Of these records 4.031 records were excluded for not meeting inclusion criteria for titles and abstracts, leaving 51 potentially relevant articles. Among these articles we identified 25 conference abstracts [16–40], 3 studies in which patients were switched between intervention and control group [41–43] and 5 studies that investigated nafamostat mesilate but not DTI versus heparin [44–48]. These 33 studies were excluded, leaving 18 studies for inclusion into data synthesis. Of note, one of these studies was provided by an author and contained unpublished data. A summary of study selection process is presented in Fig. 1.

### Study characteristics

In total 17 studies published from years 2011 to 2022 and one unpublished study were included in this meta-analysis [9–11, 49–63]. All studies had a retrospective study design and only one study was multi-center. These studies included 1.942 ECMO patients of which 1.097 patients received heparin, 703 patients received bivalirudin and 89 patients received argatroban. Of note, 55 patients received bivalirudin as secondary anticoagulation strategy, therefore their data were excluded from meta-analysis. Detailed study characteristics and definitions of outcomes are presented as (supplementary) tables. (Table 1, Table S1, Table S2).

**Table 1:** Characteristics of included studies

**Author**	**Year of publication**	**Study design**	**Type of Comparison**	**Number of participants per group**	**Adult / pediatric patients**	**Type of ECMO**	**Indication for ECMO**	**Male sex**	**Mean age ± SD (years)**	**aPTT aim (s)**
Hamzah [63]	2022 (under review)	Multi center, retrospective	Heparin vs. Bivalirudin	Total: 225 Hep.: 150 Biv.: 75	pediatric	VV-ECMO: 36 VA-ECMO: 141 eCPR: 48	eCPR: 48 CPB weaning: 115 Not reported: 62	Hep.: 74 Biv.: 38	Hep.: 8 (1, 36) Biv.: 7 (2, 37) (months, median, IQR)	Not reported
Pieri [58]	2021	Single center, retrospective	Heparin vs. Bivalirudin	Total: 125 Hep.: 26 Biv.: 99	All adult patients	VV-ECMO only	ARDS only	Total: 93	Not reported	Hep.: 55–60 Biv.:55–60
Sheridan [9]	2021	Single center, retrospective	Heparin vs. Bivalirudin	Total: 150 Hep.: 50 Biv.: 100	All adult patients	VV-ECMO: 52 VA-ECMO: 88	CS: 58 Resp. fail.: 59 PE: 10 CPB weaning: 11 Others:12	Total: 106 Hep.: 32 Biv.: 74	Total: 53 ± 14.5 Hep.: 53 ± 14 Biv.: 54 ± 15	Hep.: < 95 and anti-FXa 0.3–0.7 IU/mL Biv.:45–75
Machado [54]	2021	Single center, retrospective	Heparin vs. Bivalirudin	Total: 32 Hep.: 14 Biv.: 18	All pediatric patients	VA-ECMO: 30 VV-ECMO: 1 Hybrid: 1	Not reported	Total: 12 Hep.: 9 Biv.: 7	Hep.: 39.8 ± 76.1 Biv.: 36 ± 58.8 (months)	Not reported (individual goals)
Seelhammer [62]	2021	Single center, retrospective	Heparin vs. Bivalirudin	Total: 422 Hep.: 288 Biv.: 134	Adult: 333 Pediatric: 89	VA-ECMO: 358 VV-ECMO: 64	Post cardiotomy: 162 CS: 100 Resp. fail.: 86 eCPR: 69 Transplant: 5	Total: 265 Hep.: 183 Biv.: 82	Not reported	Hep.: 60–90 Biv.: 60–80
Schill [63]	2021	Single center, retrospective	Heparin vs. Bivalirudin vs. switched	Total: 54 Hep.: 34 Biv.: 14 Switched: 8	All pediatric patients	VA-ECMO: 38 VV-ECMO: 18	Post cardiotomy: 20 Resp. fail.: 19 CS: 17	Not reported	Hep.: 16.3 (4.8, 143.7) Biv.: 5.5 (3.7, 79.6) (months, median,IQR)	Hep.: anti-FXa 0.3–0.7 IU/mL Biv.: 60–95
Kaushik [52]	2021	Single center, retrospective	Heparin vs. Bivalirudin vs. switched	Total: 39 Hep.: 27 Biv.: 8 Switched: 4	All pediatric patients	VA-ECMO:34 VV-ECMO: 4 Hybrid: 1	Resp. Fail.: 12 CS: 11 eCPR: 6	Total: 20 Hep.: 15 Biv.: 3	Hep.: 4.0 (0.5, 92.0) Biv.: 0.6 (0.0, 80.0) (months, median, IQR)	Hep.: 60–90 Biv.: 60–90
Rivosecchi [60]	2021	Single center, retrospective	Heparin vs. Bivalirudin	Total: 295 Hep.: 162 Biv.: 133	All adult patients	VV-ECMO only	Resp. fail.: 145 Pre/post-transplant: 108 Post cardiotomy: 20 Others: 22	Total: 146 Hep.: 95 Biv.: 81	Hep.: 49 (36,61) Biv.: 49 (36,61) (median, IQR)	Hep.: anti-FXa 0.25–0.35 IU/mL Biv.: 60–75
Fisser [10]	2021	Single center, retrospective	Heparin vs. Argatroban	Total: 117 Hep.: 78 Arg.: 39	All adult patients	VV-ECMO only	ARDS only	Total: 80 Hep.: 51 Arg.: 29	Hep.: 56 (48,63) Arg.: 55 (46,61) (median, IQR)	Hep.: 45–55 Arg.:45–55
Cho [50]	2021	Single center, retrospective	Heparin vs. Argatroban	Total: 35 Hep.: 24 Arg.: 11	All adult patients	VA-ECMO: 10 VV-ECMO: 21 Hybrid: 4	Not reported	Total: 22 Hep.: 15 Arg.: 7	Total: 46 ± 17 Hep.: 45 ± 16 Arg.: 49 ± 20	Hep.: 40–60 or 60–80 (high dose) Arg.:43–85
Hamzah [11]	2020	Single center, retrospective	Heparin vs. Bivalirudin	Total: 32 Hep.: 16 Biv.: 16	All pediatric patients	VA-ECMO: 29 VV-ECMO: 3	Post cardiotomy:13 Others: not reported	Total: 14 Hep.: 8 Biv.: 6	Total: 12 (0–212) Hep.: 59 (0, 212) Biv.: 31 (0–99) (months, median, IQR)	Hep.: 60–80 Biv.: 58–78 or 50–70 (open chest)
Kaseer [51]	2020	Single center, retrospective	Heparin vs. Bivalirudin	Total: 52 Hep.: 33 Biv.: 19	All adult patients	VA-ECMO: 28 VV-ECMO: 24	CS:15 ARDS:24 Transplant: 17 Others:1	Total: 37 Hep.: 25 Biv.: 12	Total: 55 (18, 83) Hep.: 53 (21, 83) Biv.: 56 (18, 71) (median, IQR)	Hep.: 50–70 or 40–60 Biv.: 60–90 or 50–70
Macielak [55]	2019	Single center, retrospective	Heparin vs. Bivalirudin vs. switched	Total: 153 Hep.: 100 Biv.: 10 Switched: 43	All adult patients	VA-ECMO: 134 Other types not reported	Salvage: 61% CS: 46% ARDS:29% Resp. fail.: 29% CPB weaning: 23% Others: 12%	Total: 127	Total: 52.8 ± 14.2 Hep.: 51.4 ± 14.0 Biv.: 57.9 ± 13.8	Hep.: 72–95 Biv.: 60–80
Berei [49]	2018	Single center, retrospective	Heparin vs. Bivalirudin	Total: 72 Hep.: 28 Biv.: 44	All adult patients	VA-ECMO: 66 VV-ECMO: 6	CS: 51 Sepsis: 11 Resp. fail.: 4 Others: 6	Total: 47 Hep.: 18 Biv.: 29	Hep.: 55.9 ± 13.1 Biv.: 55.2 ± 15.2	Hep.: 45–65 or 65–90 Biv.: 45–65 or 65–90
Menk [56]	2017	Single center, retrospective	Heparin vs. Argatroban	Total: 78 Hep.: 39 Arg.: 39	All adult patients	VV-ECMO: 43 pECLA: 24 Hybrid: 11	ARDS only	Total: 54 Hep.: 27 Arg.: 27	Hep.: 48 (35,64) Arg.: 47 (36,60) (median, IQR)	Hep.: 50–75 Arg.:50–75
Ljajikj [53]	2017	Single center, retrospective	Heparin vs. Bivalirudin	Total: 20 Hep.: 10 Biv.: 10 (after PS-Matching)	All adult patients	VA-ECMO only	support pre, during and after LVAD implantation only	Total: 17 Hep.: 9 Biv.: 8	Hep.: 52.5 ± 9.7 Biv.: 48.2 ± 14.1	Not reported
Pieri [57]	2013	Single center, retrospective	Heparin vs. Bivalirudin	Total: 20 Hep.: 10 Biv.: 10	All adult patients	VA-ECMO: 10 VV-ECMO: 10	Not reported	Total: 16 Hep.: 9 Biv.: 7	Hep.: 54 ± 12.7 Biv.: 59.5 ± 14.4	Hep.: 45–60 Biv.: 45–60
Ranucci [59]	2011	Single center, retrospective	Heparin vs. Bivalirudin	Total: 21 Hep.: 8 Biv.: 13	Adult: 12 Pediatric: 9	VVA-ECMO: 21	Post cardiotomy only	Not reported	Hep.: 13.9 ± 19 Biv.: 36.5 ± 29	Hep.: 50–80 Biv.: 50–80

*ARDS* acute respiratory distress syndrome, *Arg* Argatroban, *Biv* Bivalirudin, *CPB* cardiopulmonary bypass, *CS* cardiogenic shock, *eCPR* extracorporeal cardio pulmonary resuscitation, *Hep* Heparin, *IQR* interquartile range, *PE* pulmonary embolism, *Resp. Fail.* respiratory failure, *VA-ECMO* venoarterial extracorporeal membrane oxygenation, *VV-ECMO* venovenous extracorporeal membrane oxygenation

### Risk of bias assessment

**Fig. 2:**
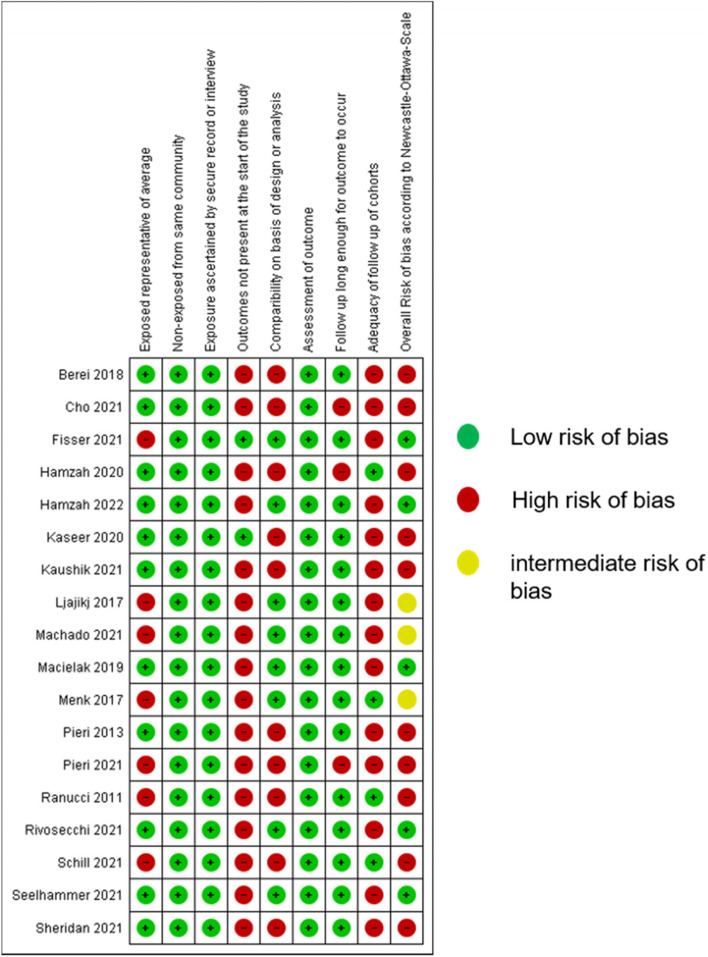
Risk of bias assessment. Legend: The figure shows risk of bias for included studies using the Newcastle Ottawa scale. Overall risk of bias is presented as low (green), intermediate (yellow) or high (red)

After assessment of risk of bias, the majority of studies (10 studies) presented a high risk of bias, 3 studies had intermediate risk and only 5 studies had low risk of bias (Fig. 2).

### Results of individual studies and data syntheses for primary and secondary outcomes

#### In-hospital mortality

**Fig. 3:**
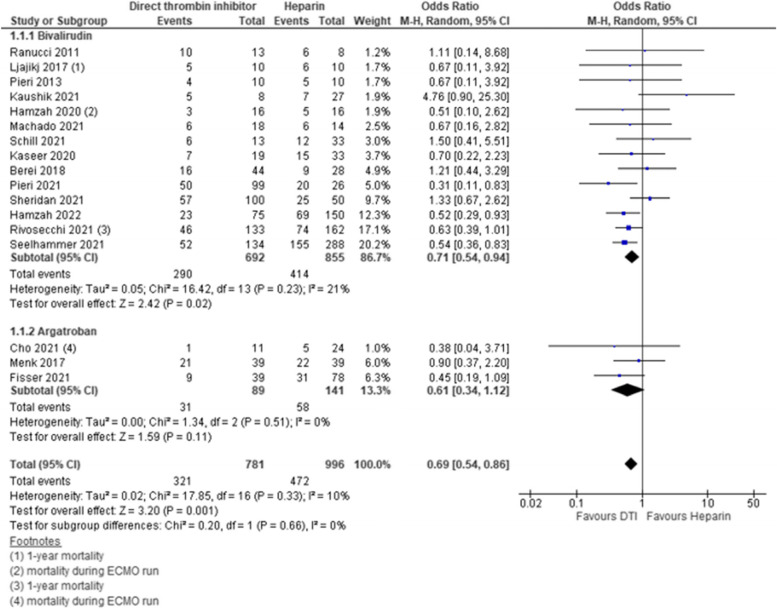
Mortality. Legend: The figure shows results of data synthesis for mortality. Pooled estimates are presented as Odds ratios for direct thrombin inhibitors versus heparin as well as for bivalirudin and argatroban subgroups

Seventeen studies reported on mortality and were included into analysis. In 14 studies, bivalirudin was compared to heparin, the remaining 3 studies compared argatroban to heparin. Four studies had a low risk of bias and contributed to analysis with a weight of 55.9%, 3 studies had intermediate risk of bias with a weight of 10.2% and 10 studies had high risk of bias with a weight of 34%. In-hospital mortality was significantly lower for DTI as compared to heparin [pooled estimate OR 0.69, 95% CI 0.54–0.86; Z = 3.20; *p* = 0.001]. Overall heterogeneity was low with I^2^ = 10% [Chi^2^ = 17.85, df = 16; *p* = 0.33]. Subgroup analysis for bivalirudin and argatroban showed significant reduction of in-hospital mortality for bivalirudin but not for argatroban as compared to heparin [bivalirudin—pooled estimate OR 0.71, 95% CI 0.54–0.94; Z = 2.42; p = 0.02; argatroban—pooled estimate OR 0.61, 95% CI 0.34–1.12; Z = 1.59; p = 0.11]. Heterogeneity measured by I^2^ within subgroups was 21% for bivalirudin and 0% for argatroban [bivalirudin—Chi^2^ = 16.42, df = 13; p = 0.23; argatroban—Chi^2^ = 1.34, df = 2; *p* = 0.51]. However, no statistical difference between subgroups was detected [Chi^2^ = 0.20, df = 1; *p* = 0.66; I^2^ = 0%]. Adult and pediatric patients both showed lower incidence of mortality with DTI as compared to heparin [pediatric—pooled estimate OR 0.65, 95% CI 0.43–0.99; Z = 2.02; *p* = 0.04; adult—pooled estimate OR 0.67, 95% CI 0.53 -0.85; Z = 3.31; *p* = 0.0009]. No heterogeneity within subgroups or subgroup differences were detected. Additionally, we explored risk of bias of studies as potential source for heterogeneity. We identified studies with high risk of bias as source for heterogeneity with I^2^ = 24% as compared to studies with low and intermediate risk of bias with I^2^ = 0% respectively. Sensitivity analysis using RR and fixed effects model did not affect these results. Estimates for each study and the subgroups are presented within the Forrest-plots (Figs. 3, S1, S2) (Table 2).

**Table 2:** Summary of findings table

Research topic: Direct thrombin inhibitors compared with heparin for extracorporeal membrane oxygenation therapy						
**Patients: Adult and pediatric patients** **Setting: In-hospital extracorporeal membrane oxygenation therapy** **Intervention: Direct thrombin inhibitors** **Comparison: Unfractionated heparin**						
**Outcomes**	**Illustrative comparative risks**^b^ **(95% CI)**		**Relative effect** **(95% CI)**	**No of Participants** **(studies)**	**Quality of the evidence** **(GRADE)**	**Comments**
	**Assumed risk**^**a**^	**Corresponding risk**				
	**unfractionated heparin**	**Direct thrombin inhibitors**				
**Mortality**	**474 per 1000**	**393 per 1000** (346 to 450)	**RR 0.83** (0.73 to 0.95)	1777 (17)	⊕ ⊝ ⊝ ⊝ **very low** due to lack of RCTs, risk of bias, publication bias	
**Major bleeding events**	**501 per 1000**	**336 per 1000** (251 to 456)	**RR 0.67** (0.5 to 0.91)	1355 (16)	⊕ ⊝ ⊝ ⊝ **very low** due to lack of RCTs, risk of bias, publication bias	
**Minor bleeding events**	**287 per 1000**	**247 per 1000** (195 to 316)	**RR 0.86** (0.68 to 1.10)	632 (8)	⊕ ⊝ ⊝ ⊝ **very low** due to lack of RCTs, risk of bias, imprecision	
**Pump-related thrombosis**	**233 per 1000**	**163 per 1000** (121 to 217)	**RR 0.7** (0.52 to 0.93)	1361 (13)	⊕ ⊝ ⊝ ⊝ **very low** due to lack of RCTs, risk of bias, imprecision, publication bias	
**Patient-related thrombosis**	**200 per 1000**	**162 per 1000** (118 to 220)	**RR 0.81** (0.59 to 1.10)	1447 (15)	⊕ ⊝ ⊝ ⊝ **very low** due to lack of RCTs, risk of bias, inconsistency, publication bias	
**length of ECMO therapy** **(hours and days)**	See comment	The SMD in length of ECMO therapy in the intervention groups was 0.12 higher (-0.03 lower to 0.27 higher)		1274 (12)	⊕ ⊝ ⊝ ⊝ **very low** due to lack of RCTs, risk of bias, imprecision, publication bias	Mean for control group not estimable as different measures were used for outcome assessment
**length of hospital stay** **(days)**	The mean time to anticoagulation goal ranged across control groups from 5 to 47 days	The SMD in length of hospital stay in the intervention groups was 0.19 higher (-0.30 lower to 0.69 higher)		467 (4)	⊕ ⊝ ⊝ ⊝ **very low** due to lack of RCTs, risk of bias, imprecision, publication bias	
**time to anticoagulation goal** **(hours)**	The mean time to anticoagulation goal ranged across control groups from 9 to 32 h	The SMD in time to anticoagulation goal in the intervention groups was 0.2 lower (-0.73 lower to 0.34 higher)		324 (4)	⊕ ⊝ ⊝ ⊝ **very low** due to lack of RCTs, risk of bias, imprecision, publication bias	
**Percentage of time within therapeutic range** **(percentage)**	The mean percentage of time within therapeutic range ranged across control groups from 11 to 31 percent	The SMD of percentage of time within therapeutic range in the intervention groups was 0.54 higher (0.14 to 0.94 higher)		491 (5)	⊕ ⊝ ⊝ ⊝ **very low** due to lack of RCTs, risk of bias, publication bias	

GRADE Working Group grades of evidence

High quality: Further research is very unlikely to change our confidence in the estimate of effect

Moderate quality: Further research is likely to have an important impact on our confidence in the estimate of effect and may change the estimate

Low quality: Further research is very likely to have an important impact on our confidence in the estimate of effect and is likely to change the estimate

Very low quality: We are very uncertain about the estimate

*CI* Confidence interval, *RR* Risk Ratio, *SMD* Standardized mean difference, *RCTs* randomized controlled trials

^a^Control group risk estimates come from pooled estimates of control groups

^b^The basis for the assumed risk (e.g. the median control group risk across studies) is provided in footnotes. The corresponding risk (and its 95% confidence interval) is based on the assumed risk in the comparison group and the relative effect of the intervention (and its 95% CI)

### Major bleeding events

**Fig. 4:**
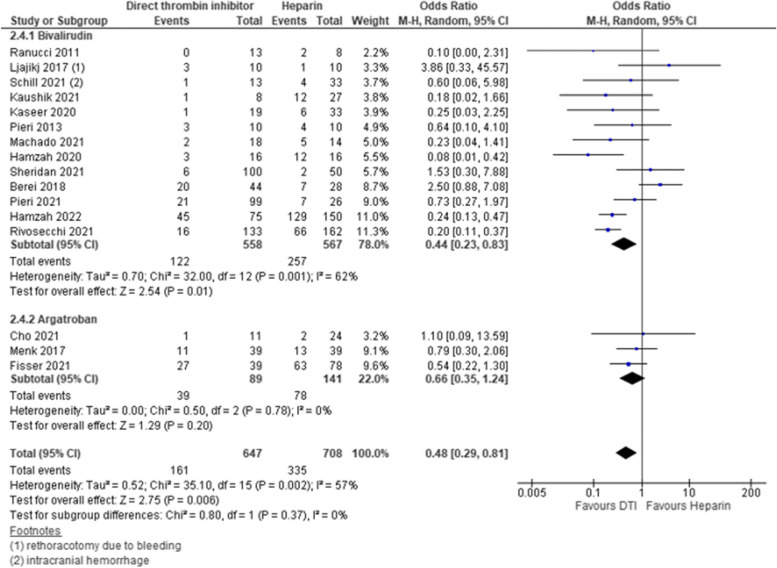
Major bleeding events. Legend: The figure shows results of data synthesis for major bleeding events. Pooled estimates are presented as Odds ratios for direct thrombin inhibitors versus heparin as well as for bivalirudin and argatroban subgroups

Fifteen studies reported on major bleeding events of which 12 studies compared bivalirudin and 3 studies compared argatroban to heparin. Three studies had low risk of bias and contributed to analysis with a weight of 32%, another 3 studies had intermediate risk of bias with a weight of 17.4% and 10 studies presented high risk of bias with a weight of 50,6%. Major bleeding was lower in DTI group as compared to heparin group [pooled estimate OR 0.48, 95% CI 0.29–0.81; Z = 2.75; *p* = 0.006] however, overall heterogeneity was high [I^2^ = 57%, Chi^2^ = 35.1, df = 15, *p* = 0.002]. Subgroup analysis revealed that major bleeding was significantly reduced for bivalirudin but not for argatroban, and in pediatric patients but not in adult patients with DTI [bivalirudin—pooled estimate OR 0.44, 95% CI 0.23–0.83; Z = 2.54; *p* = 0.01; argatroban—pooled estimate OR 0.66, 95% CI 0.35–1.24; Z = 1.29; p = 0.20; pediatric—pooled estimate OR 0.22, 95% CI 0.13–0.38; Z = 5.43; *p* =  < 0.0001; adult—pooled estimate OR 0.74, 95% CI 0.38–1.41; Z = 0.92; *p* = 0.36]. We used subgroup analysis to explore potential sources of heterogeneity and identified that heterogeneity was high between studies that investigated bivalirudin versus heparin and studies which investigated anticoagulation regime in adult patients [Bivalirudin subgroup—I^2^ = 62%, Chi^2^ = 32, df = 12, *p* = 0.001; adult subgroup—I^2^ = 63%, Chi^2^ = 24, df = 9, *p* = 0.004]. Sensitivity analysis using RR and fixed effects model did not change the overall results but use of fixed effect model additionally lead to a significant reduction in major bleeding for subgroup of adult patients with DTI by narrowing the CI [adult—pooled estimate OR 0.54, 95% CI 0.39–0.74; Z = 3.75; *p* = 0.0002]. Estimates for each study and the subgroups are presented within the Forrest-plots (Figs. 4, S3).

### Minor bleeding events

**Fig. 5:**
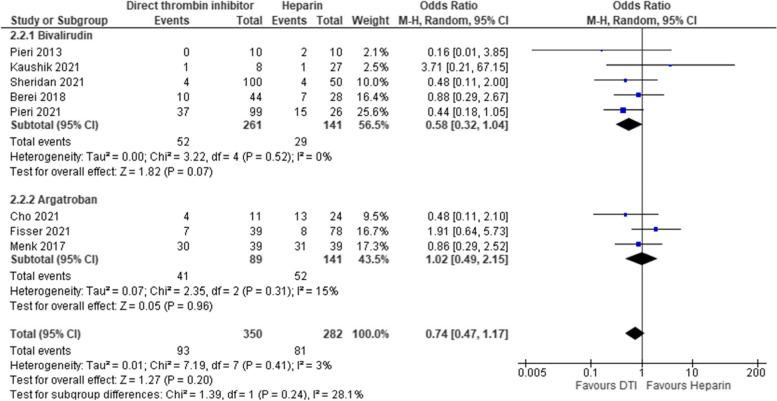
Minor bleeding events. Legend: The figure shows results of data synthesis for minor bleeding events. Pooled estimates are presented as Odds ratios for direct thrombin inhibitors versus heparin as well as for bivalirudin and argatroban subgroups

A total of 8 studies reported on minor bleeding events of which 5 studies compared bivalirudin and 3 studies compared argatroban to heparin during ECMO therapy. Overall no significant differences in minor bleeding events was detected between DTI and Heparin [pooled estimate OR 0.74, 95% CI 0.47–1.17; Z = 1.27; *p* = 0.20], Use of argatroban showed no effect on minor bleeding events as compared to heparin [pooled estimate OR 1.02, 95% CI 0.49–2.15; Z = 0.05; *p* = 0.96]. Overall heterogeneity and heterogeneity within subgroups were low [overall—I^2^ = 3%, Chi^2^ = 7.19, df = 7, *p* = 0.41; bivalirudin subgroup—I^2^ = 0%, Chi^2^ = 3.22, df = 4, *p* = 0.52; argatroban subgroup—I^2^ = 15%, Chi^2^ = 2.35, df = 2, *p* = 0.31]. Sensitivity analysis using RR and fixed effects model did not change the overall results but use of RR changed non-significant trend to a significant reduction in minor bleeding in bivalirudin patients by narrowing the CI [adult—pooled estimate RR 0.68, 95% CI 0.48–0.97; Z = 2.11; *p* = 0.04] Estimates for each study and the subgroups are presented within the Forrest-plots. (Fig. 5).

### Patient-related thrombosis

**Fig. 6:**
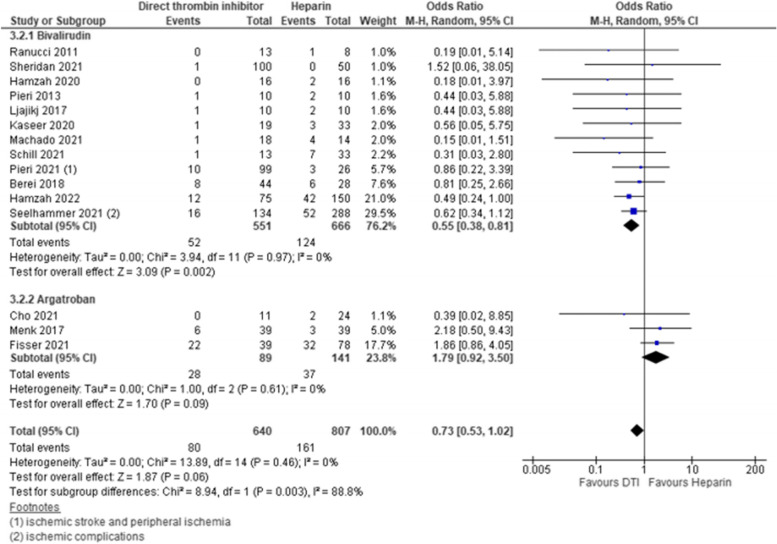
Patient-related thrombosis. Legend: The figure shows results of data synthesis for patient-related thrombotic events. Pooled estimates are presented as Odds ratios for direct thrombin inhibitors versus heparin as well as for bivalirudin and argatroban subgroups

Fifteen studies reported on patient-related thrombosis including 12 studies comparing bivalirudin and all 3 studies comparing argatroban to heparin. Overall pooled estimates indicated that use of DTI might be beneficial however, the finding was not statistically significant [pooled estimate OR 0.73, 95% CI 0.53–1.02; Z = 1.87; p = 0.06]. Subgroup analysis for anticoagulants revealed that use of bivalirudin reduces patient-related thrombosis while use of argatroban might be not beneficial as trend favored heparin [bivalirudin- pooled estimate OR 0.55, 95% CI 0.38–0.81; Z = 3.09; p = 0.002; argatroban—pooled estimate OR 1.79, 95% CI 0.92–3.50; Z = 1.70; p = 0.09]. This resulted in significant difference between subgroups [test for subgroup differences—I^2^ = 88.8%, Chi^2^ = 8.94, df = 1, *p* = 0.003]. Overall heterogeneity and heterogeneity within subgroups was not detected [overall—I^2^ = 0%, Chi^2^ = 13.89, df = 14, p = 0.46; bivalirudin subgroup—I^2^ = 0%, Chi^2^ = 3.94, df = 11, p = 0.97; argatroban subgroup—I^2^ = 0%, Chi^2^ = 1.0, df = 2, *p* = 0.61]. Use of RR did not change the results. Sensitivity analysis with fixed effects model changed the non-significant trend to significant benefit of DTI for patient-related thrombosis by narrowing the CI [pooled estimate OR 0.71, 95% CI 0.52–0.98; Z = 2.10; *p* = 0.04]. Estimates for each study and the subgroups are presented within the Forrest-plots. (Fig. 6).

### Pump-related thrombosis

**Fig. 7:**
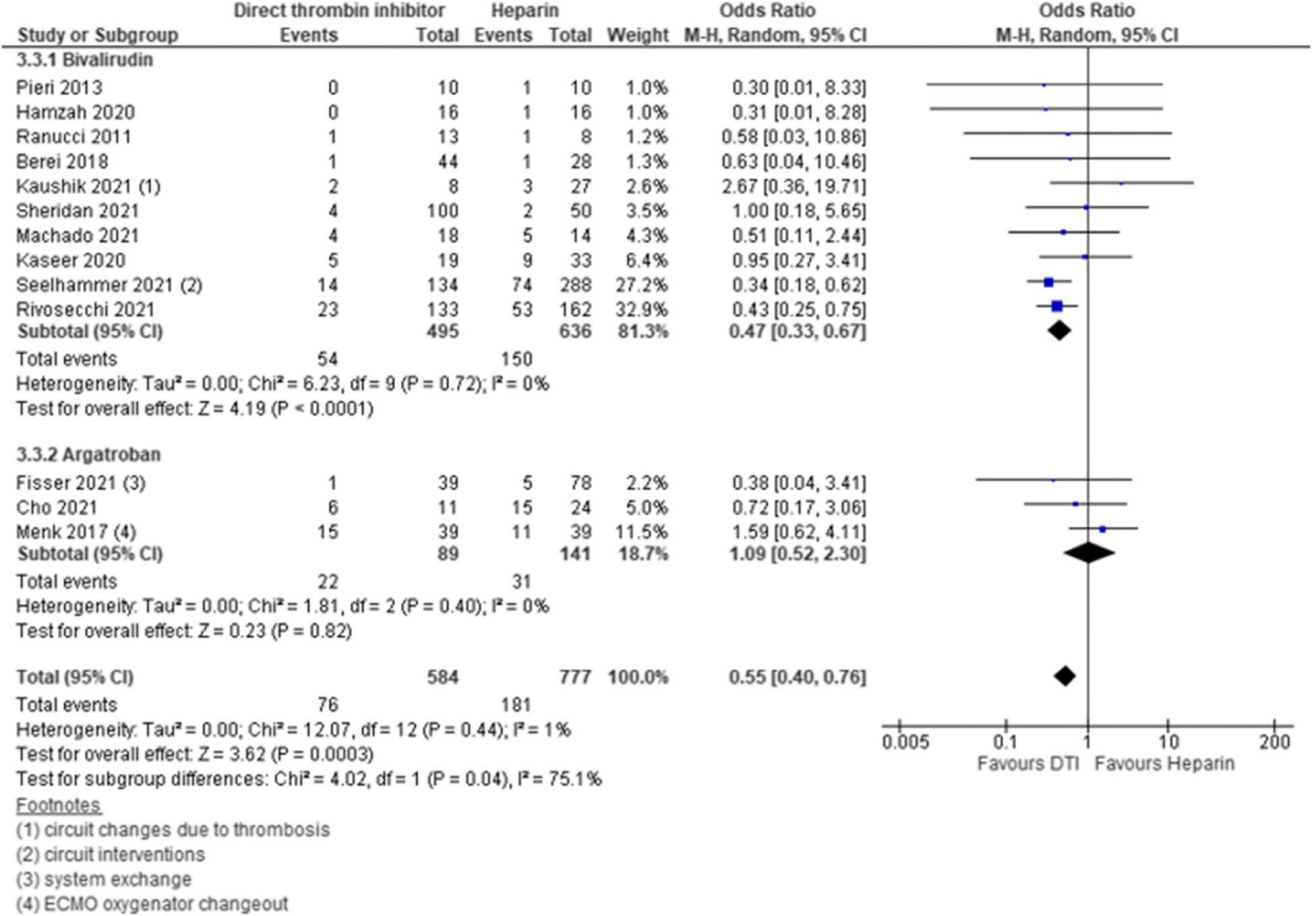
Pump-related thrombosis. Legend: The figure shows results of data synthesis for pump-related thrombotic events. Pooled estimates are presented as Odds ratios for direct thrombin inhibitors versus heparin as well as for bivalirudin and argatroban subgroups

Thirteen studies reported on pump-related thrombosis. Ten of these studies compared bivalirudin to heparin, 3 studies used argatroban as DTI. Three of these studies presented low risk of bias, 2 studies had intermediate risk of bias, and 8 studies had high risk of bias. Pump-related thrombosis occurred less frequent in DTI group as compared to heparin group [pooled estimate OR 0.55, 95% CI 0.40–0.76; Z = 3.62; *p* = 0.0003]. This finding was mainly driven by patients who received bivalirudin compared to heparin [subgroup bivalirudin—pooled estimate OR 0.47, 95% CI 0.33–0.67; Z = 4.19; *p* =  < 0.0001]. Argatroban showed no beneficial influence on occurrence of pump-related thrombosis as compared to heparin [subgroup argatroban—pooled estimate OR 1.09, 95% CI 0.52–2.30; Z = 0.23; *p* = 0.82]. Thus, significant difference between subgroups was detected [test for subgroup differences—I^2^ = 75.1%, Chi^2^ = 4.02, df = 1, p = 0.04]. However, this did not lead to overall heterogeneity [I^2^ = 1%, Chi^2^ = 12.07, df = 12, p = 0.44]. Estimates for each study and the subgroups are presented within the Forrest-plots. (Fig. 7).

### Length of ECMO therapy

**Fig. 8:**
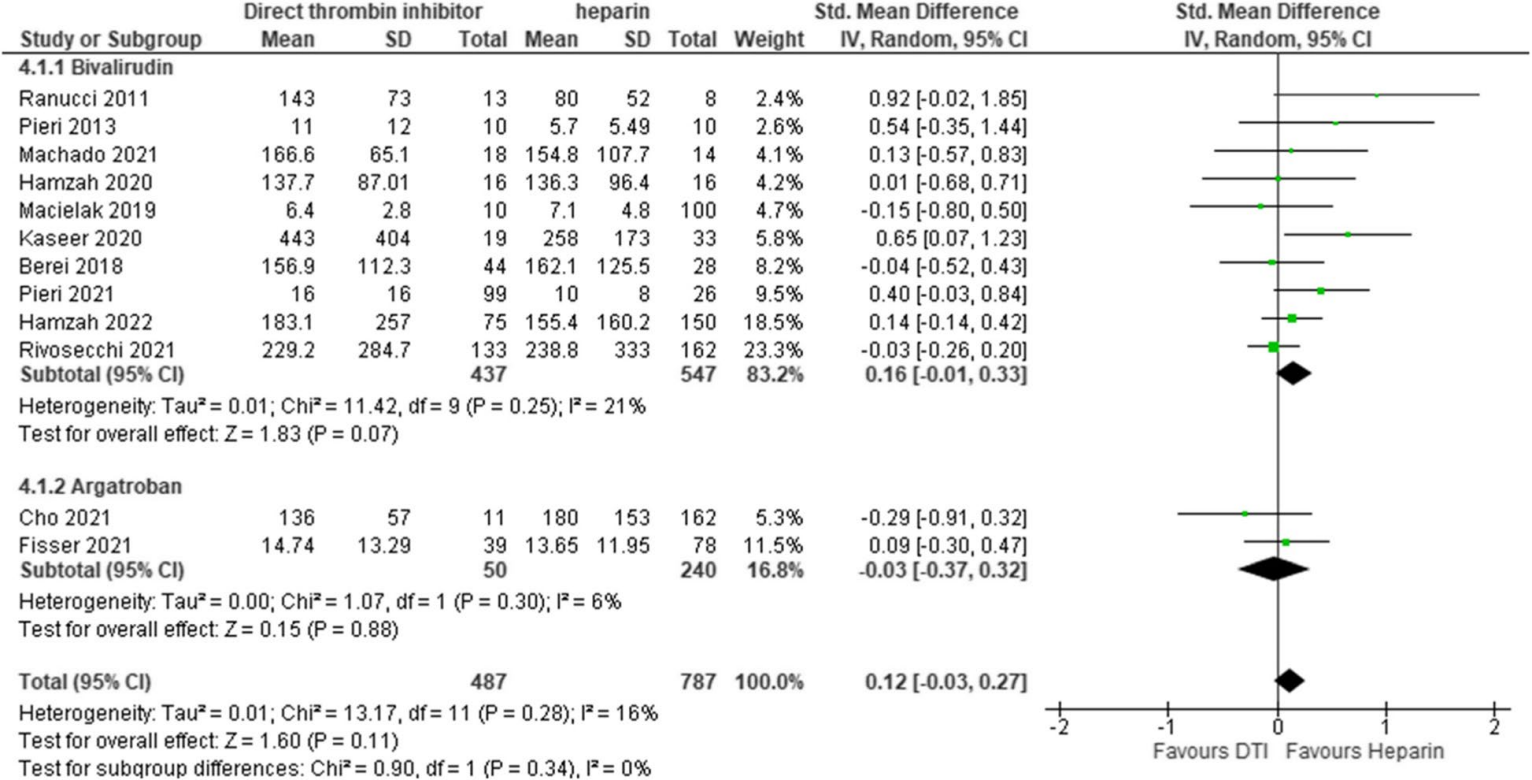
Length of extracorporeal membrane oxygenation therapy. Legend: The figure shows results of data synthesis for length of extracorporeal membrane oxygenation therapy. Pooled estimates are presented as standardized mean difference for direct thrombin inhibitors versus heparin as well as for bivalirudin and argatroban subgroups

We analyzed length of ECMO therapy between DTI and heparin patients. In total 12 studies reported on length of ECMO therapy. Ten studies compared bivalirudin to heparin and 2 studies used argatroban. Of these studies 4 studies had low risk of bias, 1 study had intermediate risk of bias and 7 studies had high risk of bias. Overall length of ECMO therapy showed no difference between DTI and Heparin [pooled estimate SMD 0.12, 95% CI -0.03–0.27; Z = 1.60; *p* = 0.11] with a moderate overall heterogeneity [I^2^ = 16%, Chi^2^ = 13.17, df = 11, *p* = 0.28]. Bivalirudin subgroup was detected as possible source for heterogeneity [I^2^ = 21%, Chi^2^ = 11.42, df = 9, *p* = 0.25]. Use of fixed effects model and Mean difference did not change the results in sensitivity analysis. Estimates for each study and the subgroups are presented within the Forrest-plots. (Fig. 8).

### Percentage of time within therapeutic range

Only 5 studies reported on percentage of time within therapeutic range during ECMO therapy. All studies compared bivalirudin to heparin for ECMO therapy. Among these studies 2 had low risk of bias, 1 study had intermediate risk of bias and 2 studies had high risk of bias. Overall pooled estimate indicated that patients with DTI during ECMO had higher percentage of time within therapeutic range [pooled estimate SMD 0.54, 95% CI 0.14–0.94; Z = 2.65; p = 0.008]. However, heterogeneity was high between studies [I^2^ = 67%, Chi^2^ = 12.12, df = 4, *p* = 0.02]. Subgroup analysis for risk of bias revealed that studies with low risk of bias showed no heterogeneity [I^2^ = 0%, Chi^2^ = 0.07, df = 1, *p* = 0.79] but heterogeneity was present in studies with intermediate and high risk of bias [I^2^ = 57%, Chi^2^ = 4.63, df = 2, *p* = 0.1]. Sensitivity analysis changed results for adult patients by using fixed effects model, overall result was not affected [ adult—pooled estimate SMD 0.74, 95% CI 0.47–1.01; Z = 5.42; *p* =  < 0.0001] (Fig. S4).

### Length of hospital stay and time to anticoagulation goal

Only 4 studies reported for length of hospital stay and time to anticoagulation goal respectively. No difference could be detected between DTI and heparin patients. Additional information and Forrest-plots are attached as supplementary figures (Figs. S5, S6).

### Evaluation of reporting biases

We evaluated publication bias by creating funnel plots for each outcome. By visual inspection we detected relevant asymmetry of funnel plots for all outcomes beside of minor bleeding events. To reduce reporting bias, we contacted authors to contribute additional information as not all studies reported for all outcomes. However, only 4 authors responded to our request and added additional data for analysis (Fig. S7).

### Certainty of evidence

We assessed the certainty of evidence for each outcome using the GRADE approach. For every outcome certainty of evidence was judged as very low, mainly resulting from lack of randomized controlled trials and high risk of bias as well as high risk for reporting bias. (Table 1).

## Discussion

This systematic review and meta-analysis investigated the effects of DTI versus heparin on clinical outcomes in patients undergoing ECMO. The main finding of this analysis is that the use of DTI for anticoagulation is significantly associated with reduced in-hospital mortality in both pediatric and adult ECMO patients compared to heparin. In addition, DTI (especially bivalirudin) are superior to heparin in terms of major bleeding events as well as patient and pump-related thrombotic complications in our analysis. Furthermore, DTI provide a stable anticoagulation during ECMO as measured by percentage of time within therapeutic range.

### Existing literature in this field

To date, three meta-analyses are available that compared bivalirudin and heparin in patients undergoing ECMO while no meta-analysis is available for argatroban [12–14, 64]. All of the bivalirudin analyses were published in 2022 which clarifies the high relevance of this topic. We will discuss the results in the following to put our own findings in context.

Di-Huan Li and colleagues selected ten articles for their meta-analysis including 997 ECMO patients. For the primary endpoint in-hospital mortality, seven studies including 670 patients (bivalirudin group = 242 patients) remained. Based on a heterogeneity of I2 = 15%, the authors report that there was no significant difference between bivalirudin treated patients and patients receiving heparin regarding in-hospital mortality (OR = 0.81, 95%CI [0.54, 1.22], *P* = 0.32). However, subgroup analyses based on patient characteristics revealed potential survival benefit for adults (OR = 0.65, 95%CI [0.44, 0.95], *P* = 0.03). In pediatric ECMO patients, there was no significant difference in terms of survival (OR = 1.30, 95%CI [0.47, 3.56], *P* = 0.61). Regarding secondary outcomes, the analysis by Li et al. revealed that there was a significantly lower incidence of thrombosis in the bivalirudin group (OR = 0.53, 95%CI [0.36, 0.79], *P* = 0.002). Major bleeding events and ECMO duration showed no significant difference. The differences to our findings might be explained by the limited number of included studies (in total 9 studies versus 15 bivalirudin studies in our analysis). As all studies had a retrospective design and investigated rather small cohorts, even small differences regarding design, study population, intervention or endpoint definitions may account for relevant changes regarding the results. This underlines the urgent need for prospective trials. The authors also performed an analysis of cost-effectiveness which showed that the use of bivalirudin did not result in higher costs [64]. Unfortunately, only three studies comparing the cost difference between bivalirudin and heparin were available. As all data were presented as median (minimum–maximum or 25–75 percentile), a pooled meta-analysis could not be performed. This aspect remains to be investigated in future studies.

The second available meta-analysis by Mei-Juan Li and colleagues included 9 studies (= 994 patients). The authors also found a survival benefit for the bivalirudin group in adult ECMO patients (risk ratio: 0.82, 95% CI 0.69–0.99). Additionally, the use of bivalirudin was associated with reduced major bleeding events (risk ratio: 0.32, 95% confidence interval [CI] 0.22–0.49), reduced incidences of ECMO in-circuit thrombosis (risk ratio: 0.57, 95% CI 0.43–0.74) and stroke (RR: 0.52, 95% CI 0.29–0.95) and higher survival rates until weaning from ECMO (RR: 1.18, 95% CI 1.03–1.34). Of note, the authors performed a „leave-one-out “ sensitivity analysis which showed that the results for in-hospital-mortality, stroke and survival until ECMO weaning should be interpreted carefully and more prospective / good-quality studies are needed [13].

Finally, there is a third meta-analysis by Liyao Liu and colleagues which is the largest of these three as 14 studies with a total of 1501 adult and pediatric patients were included into analysis. The endpoints of interest in this study were in-hospital mortality, ECMO survival, thrombotic events, major bleeding and in-circuit thrombosis. Similar to the other meta-analyses, in-hospital-mortality was significantly lower in the bivalirudin group (OR = 0.78, 95% CI [0.61–0.99], *p* = 0.04). Furthermore, patients receiving bivalirudin for anticoagulation had significantly improved results for all other clinical outcomes (ECMO survival rate: OR = 1.50, 95% CI [1.04–2.16], *p* = 0.032; thrombotic events: OR = 0.61, 95% CI [0.45–0.83], *p* = 0.002; major bleeding: OR = 0.36, 95% CI [0.14–0.91], *p* = 0.031; in-circuit thrombosis: OR = 0.44, 95% CI [0.31–0.61], *p* = 0.000) [12].

Referring to argatroban, no meta-analysis comparing argatroban with heparin in ECMO patients is currently available. However, there is one systematic review by Geli and colleagues dealing with this topic. A total of 13 studies could be identified that investigated the use of argatroban for anticoagulation in ECMO patients. Notably, 9 out of these 13 studies were only case series which were not included into the present meta-analysis. Based on their literature review, the authors conclude that major bleeding events as well as thrombotic complications seem to be comparable between argatroban-treated patients and heparin-treated patients. However, no formal analysis was conducted [14].

### What does our analysis add to the existing literature?

Based on the existing evidence, the present analysis adds multiple new aspects to the field of anticoagulation strategies in patients undergoing ECMO. First and most importantly, we did not only focus on one specific drug (bivalirudin or argatroban), but performed an anaylsis for DTI versus heparin in general. Of course, we were also able to perform separate analyses for both drugs alone, but from a clinical perspective, the comparison seems to be suitable as both substances are following the same pharmacological target. Second, our analysis has the largest number of included studies (18 studies, 1942 patients) so far. With regard to the increasing number of ECMO-treated patients worldwide, the topic is of high relevance so that updated data are urgently needed. This aspect is even more important referring to the fact that the quality of the existing studies is low as only retrospective data are available. Thus, the addition of only one or two (good-quality) studies might be enough to change the results completely. Against this background, it is a strength of our analysis that we could include a first multicenter study that was not included into the existing meta analyses. Third, our study analyzed new endpoints that have not been investigated yet. Importantly, clinicians probably will not base their decision on the anticoagulation regimen solely on mortality data and it is essential to focus on further endpoints. Therefore, next to the established endpoints of interest (mortality, bleeding, thrombosis etc.), we also included length of ECMO support, length of hospital stay, percentage of activated partial thromboplastin time (aPTT) within therapeutic window and hours to therapeutic aPTT levels as secondary outcomes. E.g. it is a new finding that patients receiving bivalirudin were significantly longer within the therapeutic range for anticoagulation (SMD = 0.54, 95% CI [0.14–0.94], *p* = 0.008) which might be an explanation why bleeding complications and thrombotic complications were significantly reduced in these patients. However, only five studies were available for this analysis so that these findings should be interpreted with caution. The time until the therapeutic window was reached was also lower in the bivalirudin group, although these results (based on four studies) were not statistically significant. Length of hospital stay and length of ECMO therapy showed no significant differences between the two groups. Though, there was a non-significant trend for longer ECMO therapy in the bivalirudin group. This observation might be related to the fact that mortality during ECMO therapy was lower in these patients. Fourth, our analysis differentiated between minor and major bleeding events as well as between patient-related and pump-related thrombotic complications. Interestingly, the use of bivalirudin was more protective in terms of major bleeding events (OR: 0.5, 95% CI [0.30–0.85]. This finding suggests that bivalirudin might be a suitable and safe alternative even in high-risk patients for bleeding complications. Fifth, and finally, this is the first analysis comparing heparin and argatroban. While the use of bivalirudin was clearly associated with improved clinical outcomes, argatroban alone was not superior, but rather comparable to the standard therapy heparin for most endpoints. Importantly, only three studies comparing heparin and argatroban could be included. Therefore, our results might serve as a first insight, but transferability of these data must be regarded as very limited.

### Strengths and limitations

This was a preplanned, protocol-based analysis, of four large electronic medical libraries. In total we detected 18 relevant articles. We enrolled a large number of ECMO patients in this meta-analysis and added new information to the existing literature. Despite promising results this meta-analysis has some limitations. Due to the lack of randomized controlled trials which introduces high risk of bias, certainty in our findings must be regarded as very limited. We tried to address reporting bias by contacting authors and requesting additional data for analysis as not all studies reported for every outcome. However, only four authors responded to our request and therefore a majority of data could not be included into our analysis. Of note we were able to include unpublished data of a multicenter retrospective study which complements the existing data in this field. Another limitation of this study is that the definitions of secondary outcomes (e.g. minor / major bleeding or patient and pump related thrombosis) may be different in the included studies. To ensure more transparency, the exact definitions of relevant secondary outcomes are presented in table S2. Furthermore, there might be several other important factors clinicians might consider when deciding about the choice of anticoagulation. As mentioned in the discussion, mortality data alone probably will not be sufficient and although several secondary endpoints have been investigated, multiple other factors are still lacking. In particular, there are no data on more patient-centered outcomes such as life impact or quality of life which becomes more and more important in the setting of mechanical circulatory support. Additionally, center effects, publication bias or reporting bias have to be considered when interpreting the results. Finally, although comparing two DTI is a strength of this study, this may also be regarded as a limitation as the information gathered is only through comparing them via heparin as an intermediary which limits this comparison.

## Conclusions

In conclusion, the present meta-analysis revealed that the use of DTI for anticoagulation in patients undergoing ECMO is associated with reduced in-hospital mortality as well as a reduced incidence of major bleeding and thrombotic events. Especially the use of bivalirudin showed positive effects on these outcomes in comparison with the standard therapy heparin. Before drawing final conclusions if DTI are really superior to the standard therapy heparin, well designed prospective (randomized) studies are urgently needed. Until these data are available, DTI may at least be regarded as a safe, effective and potentially beneficial strategy for anticoagulation in this cohort.

## Supplementary Information

**Additional file 1: Figure S1.** Mortality analysisfor adult and pediatric patients. **FigureS2.** Mortality analysis for risk of bias. **Figure S3.** Major bleeding events for adult and pediatric patients. **Figure S4.** Percentage of time withintherapeutic range. **Figure S5.** lengthof hospital stays. **Figure S6.** Timeto reach anticoagulation goal. **Figure S7.**Funnel plots.

**Additional file 2.** Supplementary materials 1 protocol.

**Additional file 3.** Supplementary materials 2 search strategies.

**Additional file 4: Table S1.** Detailedcharacteristics of included studies.

**Additional file 5: Table S2.** Definitions of outcomes.

## Data Availability

All data used for analysis is available in supplementary table S1. Further data is available from the corresponding author on reasonable request.

## Abbreviations

aPTT: Activated partial thromboplastin time
CS: Cardiogenic shock
DTI: Direct thrombin inhibitors
ECMO: Extracorporeal membrane oxygenation
OR: Odds ratio
RR: Risk ratio
SMD: Standardized mean difference
