# Supplementary material for: Evaluation of clinical outcomes in patients treated with heparin or direct thrombin inhibitors during extracorporeal membrane oxygenation: a systematic review and meta-analysis
Source: Thromb J. 2022 Jul 28;20:42. doi: 10.1186/s12959-022-00401-2 (PMC9330661; doi:10.1186/s12959-022-00401-2)
Supplement: Supplementary file 1 — Additional file 1: Figure S1. Mortality analysisfor adult and pediatric patients. FigureS2. Mortality analysis for risk of bias. Figure S3. Major bleeding events for adult and pediatric patients. Figure S4. Percentage of time withintherapeutic range. Figure S5. lengthof hospital stays. Figure S6. Timeto reach anticoagulation goal. Figure S7.Funnel plots. [file 12959_2022_401_MOESM1_ESM.docx]

**Supplementary figures:**

**Figure S1: Mortality analysis for adult and pediatric patients**

**
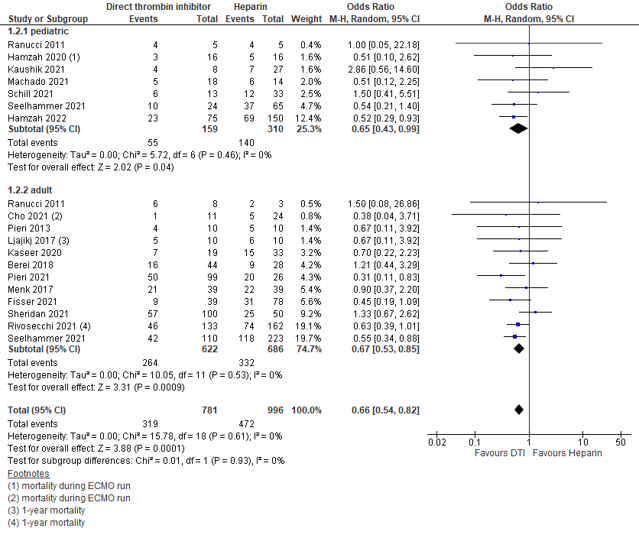
**

**Legend:** The figure shows results of data synthesis for mortality. Pooled estimates are presented as Odds ratios for direct thrombin inhibitors versus heparin in adult and pediatric patients.

**Figure S2: Mortality analysis for risk of bias**

**
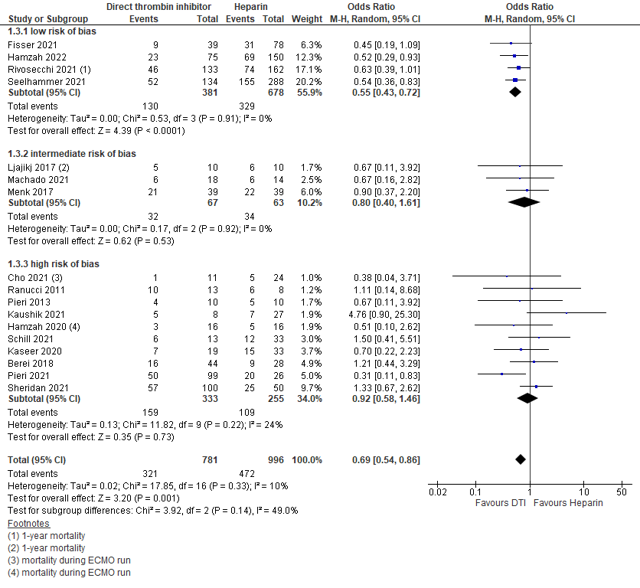
**

**Legend:** The figure shows results of data synthesis for mortality. Pooled estimates are presented as Odds ratios for direct thrombin inhibitors versus heparin in studies with different risk of bias.

**Figure S3: Major bleeding events for adult and pediatric patients**

**
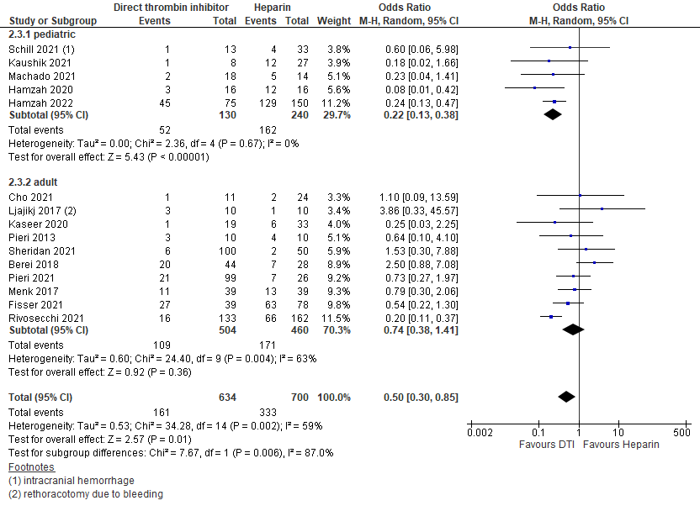
**

**Legend:** The figure shows results of data synthesis for major bleeding events. Pooled estimates are presented as Odds ratios for direct thrombin inhibitors versus heparin in adult and pediatric patients.

**Figure S4: Percentage of time within therapeutic range**

**
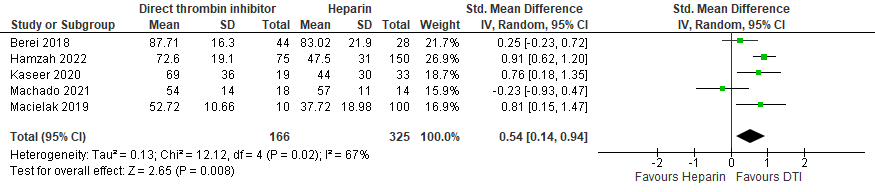
**

**Legend:** The figure shows results of data synthesis for percentage of time within therapeutic range. Pooled estimates are presented as standardized mean difference for direct thrombin inhibitors versus heparin.

**Figure S5: length of hospital stays**

**
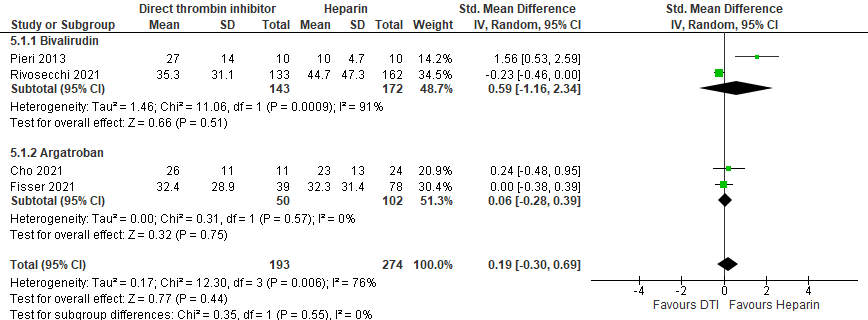
**

**Legend:** The figure shows results of data synthesis for length of hospital stays. Pooled estimates are presented as standardized mean difference for direct thrombin inhibitors versus heparin as well as for argatroban and bivalirudin subgroups.

**Figure S6: time to reach anticoagulation goal**

**
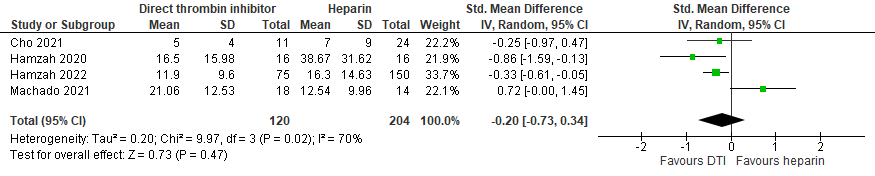
**

**Legend:** The figure shows results of data synthesis for time to reach anticoagulation goal. Pooled estimates are presented as standardized mean difference for direct thrombin inhibitors versus heparin.

**Figure S7: Funnel plots**

**
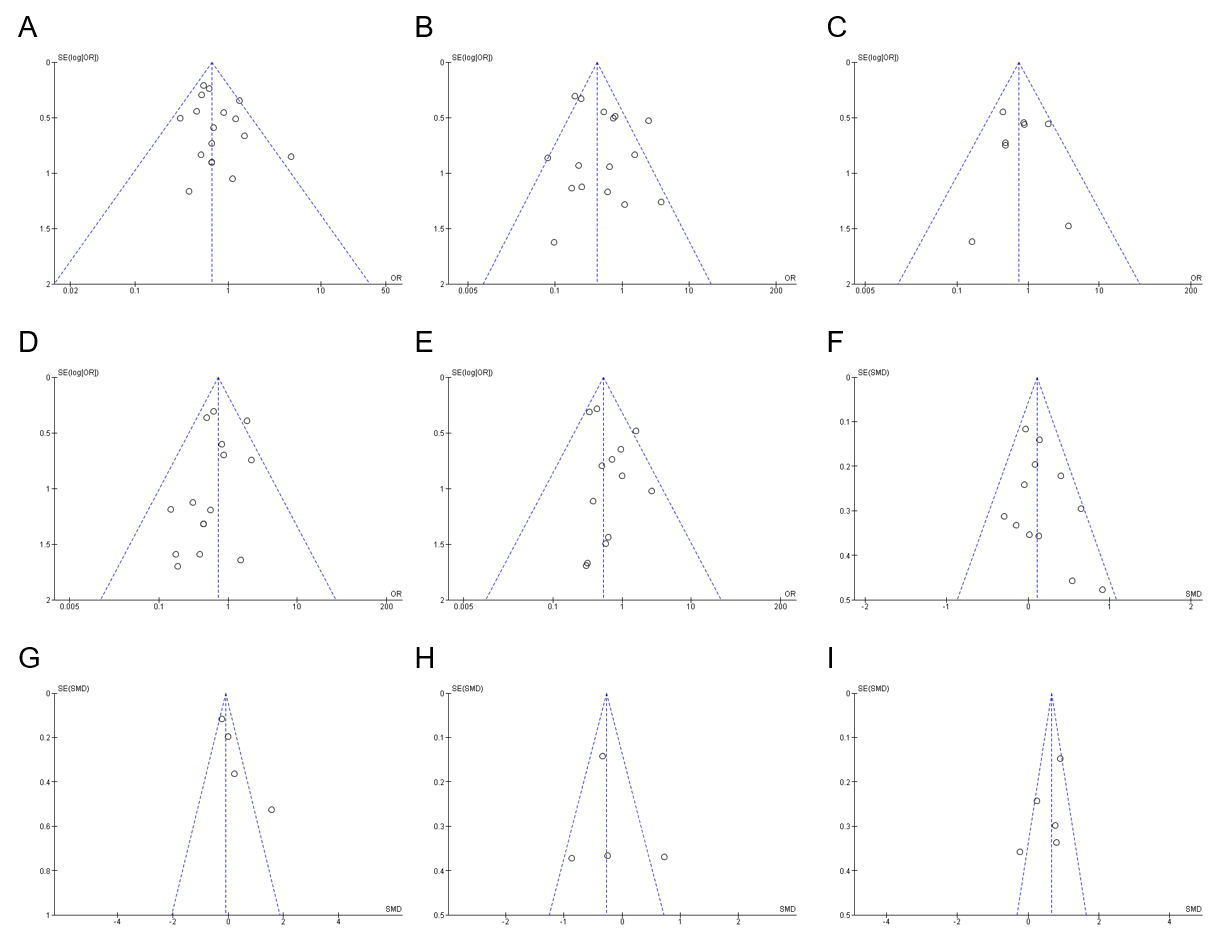
**

**Legend:**  The figure shows funnel plots for following outcomes: Mortality (A), major bleeding (B), minor bleeding (C), patient related thrombosis (D), pump related thrombosis (E), length of extracorporeal membrane oxygenation therapy (F), length of hospital stay (G), time to reach anticoagulation goal (H) and percentage of time within therapeutic range (I).
