# Supplementary material for: Evaluation of clinical outcomes in patients treated with heparin or direct thrombin inhibitors during extracorporeal membrane oxygenation: a systematic review and meta-analysis
Source: Thromb J. 2022 Jul 28;20:42. doi: 10.1186/s12959-022-00401-2 (PMC9330661; doi:10.1186/s12959-022-00401-2)
Supplement: Supplementary file 2 — Additional file 2. Supplementary materials 1 protocol. [file 12959_2022_401_MOESM2_ESM.docx]

**Protocol: Evaluation of clinical outcomes in patients with ECMO treated with Heparin or direct thrombin inhibitors: A systematic review and meta-analysis.**

**1) Background:**

Therapeutic use of extracorporeal membrane oxygenation (ECMO) devices is continuously increasing as experience and evidence with this therapeutic modality has grown during the past decade. Frequent indications for ECMO therapy are cardiogenic shock, respiratory failure, severe sepsis or postcardiotomy syndrome[1, 2]. During ECMO therapy, the exposure to exogenous surfaces and altered blood flow leads to activation of the coagulation cascade. Therefore, therapeutic anticoagulation regimens are crucial during ECMO therapy. However, not only thrombotic but also bleeding complications are frequent in these patients [3]. According to established protocols [4], most centers use heparin as the anticoagulant of choice for ECMO. Nonetheless, heparin induced thrombocytopenia and heparin resistance are conditions frequently requiring the use of alternative anticoagulants. In this context, some centers primarily use direct thrombin inhibitors (DTI) as therapeutic anticoagulation [5]. This concepts are especially used in pediatric ECMO as heparin resistance is frequent in these patients. Sanfilippo et al recently summarized the few retrospective studies reporting on the use of bivalirudin during ECMO. With regard to bleeding or thrombotic complications findings were inconclusive. Further, they did not specifically compare clinical outcomes of bivalirudin against heparin [6]. Other systematic reviews compared Bivalirudin or Argatroban to heparin in ECMO patients [7, 8]. In conclusion feasibility of alternative anticoagulant regimes others than heparin have been demonstrated for ECMO patients. However, optimal anticoagulation strategy during ECMO therapy remains unclear. Especially comparison of evidence between different DTIs versus heparin has not been demonstrated. Therefore, this systematic review will focus on clinical comparison of different DTI anticoagulation regimes compared to heparin in ECMO patients.

**2) Methods:**

**Search strategy:** We will search the following electronic databases of “PubMed/Medline”, “Cochrane Library”, “CINAHL”, and “EMBASE” and select eligible studies. Additionally, the local medical library of the university of Duesseldorf will be searched for articles. A set of MeSH (Medical Subject Headings) search terms will be used. Terms will include amongst others: “adverse drug event“, “thromboembolism“, bleeding“, “death“, “mortality”, “complication“, “thrombin antagonists”, “direct thrombin inhibitors”, “bivalirudin“, “argatroban”, “lepirudin”, “heparin”, “anticoagulant”, “circulation“, “extracorporeal“, “ECMO“ and “extracorporal membrane oxygenation“. Detailed search terms will be reported.

**3) Study selection:**

Studies comparing DTIs against heparin in pediatric or adult patients undergoing treatment with veno-arterial (VA) or veno-venous (VV) ECMO, independent of indication, will be included. Prospective and retrospective observational cohort studies as well as randomized controlled trials will be included. The search will be restricted to the English language. Studies will be excluded if:

1) not reporting any of the following endpoints: number of bleeding events, number of thromboembolic complications or mortality, length of ECMO therapy, length of hospital stay, time to achieve therapeutic aPTT, percentage of aPTT within therapeutic range.

2) Studies are systematic reviews, meta-analysis or case reports.

3) if no full scientific report is available and therefore risk of bias assessment is not applicable (congress abstracts or poster presentations).

4) if studies do not compare DTIs with heparin in ECMO patients

5) if a substantial number of patients were switched between intervention and control group but no separate group containing these patients was formed for analysis.

**4) Quality assessment:** Risk of bias will be examined separately by two independent investigators using the Newcastle-Ottawa-Scale for non-randomized trials [9]. Eligible randomized trials will be assessed by using Cochrane risk-of-bias tool for randomized trials (RoB 2). Study quality will be determined as good quality, fair quality or poor quality according to scale ratings. Publication bias will be assessed using a funnel plot.

**5) Data extraction and data synthesis:** We will first conduct a systematic review of summary data. Our primary outcome will be comparing in-hospital mortality after ECMO between DTI and heparin groups. Secondary outcomes will be minor and major bleeding events (as defined by the Extracorporeal Life support Organization [8]), patient or device related thrombotic complications (myocardial infarction, ischemic stroke, deep vein thrombosis, pulmonary artery embolism, oxygenator clotting, clotting of ECMO cannulas) during ECMO support, length of ECMO therapy, length of hospital stay, time to achieve therapeutic aPTT and percentage of aPTT within therapeutic range. To minimize reporting bias amongst included studies, first and corresponding authors will be contacted via email if reporting on any of these outcomes is unclear or not retrievable from publication.

First, data will be extracted from the full texts of publications of included studies for summary data meta-analysis. Data (if reported) will include: 1. general study information (author, study design, year of publication, recruitment period, number of included patients, name of anticoagulants compared) 2. patient baseline data (gender, age, ECMO indication, types of ECMO) and 3. Outcomes (mortality, bleeding events, thrombotic events, duration of mechanical support, length of hospital stay). A descriptive summary of the included studies will be presented as tables. If applicable, outcomes of interest of all studies will be pooled for meta-analysis. Outcomes of heparin group will be compared to DTI group using random effect models. Results will be presented as forest plots to visualize the effect and weight of each study. Heterogeneity between studies will be determined as I² and Cochrane Q.

To explore reasons for heterogeneity subgroup analysis will be performed. In this case we hypothesize that differences between adult and pediatric patients, as well as the use of different DTIs might introduce heterogeneity between studies. If feasible, this will be explored by subgroup analysis for our primary and secondary outcomes. Additionally, subgroup analysis for different risk of bias will be conducted to explain heterogeneity between studies.

**6) Process:** This systematic review will be registered at PROSPERO. The search will be conducted using databases mentioned above. Assessment of bias will be prepared manually for each study. Statistical analysis will be conducted in IBM SPSS© software version 22.0 (Armonk, NY, USA) and Review Manager (RevMan) [Computer program] Version 5.4. (The Cochrane Collaboration, 2020). Data output will be presented as Forest plots and tables extracted from Review Manager. Endnote software version X9.2 (Clarivate analytics, USA) will be used as bibliographic software. Findings of this review and meta-analysis will target a broad audience of clinicians in intensive care medicine. Hence, we aim to publish these findings in a peer-reviewed medical journal.

**7) Timetable:** Duration of full review will likely take 1-2 years. After having established this protocol, the review will be registered at PROSPERO March 2021. After submission of registration, the search and study selection will be conducted according to this protocol until August 2021. Afterwards, quality assessment and data extraction will be conducted until December 2021. Authors of included studies will be contacted for further information, as mentioned before. Data analysis and writing process will be finished by April 2022 and we aim to complete publication of this review and meta-analysis in August 2022.

**8) Citations:**

1. Paolone, S., *Extracorporeal Membrane Oxygenation (ECMO) for Lung Injury in Severe Acute Respiratory Distress Syndrome (ARDS): Review of the Literature.* Clin Nurs Res, 2017. **26**(6): p. 747-762.

2. Napp, L.C., C. Kuhn, and J. Bauersachs, *ECMO in cardiac arrest and cardiogenic shock.* Herz, 2017. **42**(1): p. 27-44.

3. Murphy, D.A., et al., *Extracorporeal membrane oxygenation-hemostatic complications.* Transfus Med Rev, 2015. **29**(2): p. 90-101.

4. Colman, E., et al., *Evaluation of a heparin monitoring protocol for extracorporeal membrane oxygenation and review of the literature.* J Thorac Dis, 2019. **11**(8): p. 3325-3335.

5. Hamzah, M., et al., *Evaluation of Bivalirudin As an Alternative to Heparin for Systemic Anticoagulation in Pediatric Extracorporeal Membrane Oxygenation.* Pediatr Crit Care Med, 2020. **21**(9): p. 827-834.

6. Sanfilippo, F., et al., *Bivalirudin for Alternative Anticoagulation in Extracorporeal Membrane Oxygenation: A Systematic Review.* J Intensive Care Med, 2017. **32**(5): p. 312-319.

7. Geli, J., et al., *Argatroban Anticoagulation for Adult Extracorporeal Membrane Oxygenation: A Systematic Review.* J Intensive Care Med, 2021: p. 885066621993739.

8. Li, D.H., et al., *Is bivalirudin an alternative anticoagulant for extracorporeal membrane oxygenation (ECMO) patients? A systematic review and meta-analysis.* Thromb Res, 2021. **210**: p. 53-62.

9. Wells GA, S.B., O’Connell D, Peterson J, Welch V, Losos M, et al., *The Newcastle-Ottawa Scale (NOS) for assessing the quality of nonrandomised studies in meta-analyses.* 2009.

8. https://www.elso.org/Portals/0/Files/elsoanticoagulationguideline8-2014-table-contents.pdf
