# Supplementary material for: Evaluation of clinical outcomes in patients treated with heparin or direct thrombin inhibitors during extracorporeal membrane oxygenation: a systematic review and meta-analysis
Source: Thromb J. 2022 Jul 28;20:42. doi: 10.1186/s12959-022-00401-2 (PMC9330661; doi:10.1186/s12959-022-00401-2)
Supplement: Supplementary file 3 — Additional file 3. Supplementary materials 2 search strategies. [file 12959_2022_401_MOESM3_ESM.docx]

**Pubmed:**

**Final search term:**

("Extracorporeal Membrane Oxygenation"[Mesh] OR “Extracorporeal membrane oxygenation”[tw] OR “extracorporeal life support”[tw] OR ECMO[tw] OR ECLS[tw] OR “veno-arterial ECMO”[tw] OR “veno-venous-ECMO”[tw] OR “VA-ECMO”[tw] OR “VV-ECMO”[tw] OR “venoarterial ECMO”[tw] OR “venovenous ECMO”[tw] OR “veno-arterial extracorporeal membrane oxygenation”[tw] OR “veno-venous extracorporeal membrane oxygenation”[tw] OR “venoarterial extracorporeal membrane oxygenation”[tw] OR “venovenous extracorporeal membrane oxygenation”[tw]) AND ("Heparin"[Mesh] OR "Antithrombins"[Mesh] OR "Anticoagulants"[Mesh] OR "Hirudins"[Mesh] OR "Peptide Fragments"[Mesh] OR "Recombinant Proteins"[Mesh] OR "Guanidines"[Mesh] OR “Bivalirudin*”[tw] OR Argatroban[tw] OR Lepirudin[tw] OR Nafamostat[tw] OR “Nafamostat mesylate”[tw] OR anticoagulation[tw] OR anticoagulant[tw] OR “Direct thrombin inhibitor*”[tw] OR DTI[tw] OR Heparin[tw]) AND ("Blood Coagulation"[Mesh] OR "Embolism and Thrombosis"[Mesh] OR "Hemorrhage"[Mesh] OR "Death"[Mesh] OR "Mortality"[Mesh] OR "Survival"[Mesh] OR "Drug-Related Side Effects and Adverse Reactions"[Mesh] OR “adverse drug event”[tw] OR “thromboembol*”[tw] OR complication[tw] OR “adverse events”[tw] OR bleeding[tw] OR “bleeding events”[tw] OR “bleeding complications”[tw] OR hemorrhage[tw] OR death[tw] OR mortality[tw] OR survival[tw])

**Search for Embase:**

Term Concept #1: (‘extracorporeal therapy device’/exp OR ‘extracorporeal membrane oxygenation device’/exp OR ‘Extracorporeal membrane oxygenation’:ti,ab,kw,de OR ‘extracorporeal life support’:ti,ab,kw,de OR ‘ECMO’:ti,ab,kw,de OR ‘ECLS’:ti,ab,kw,de OR ‘veno-arterial ECMO’:ti,ab,kw,de OR ‘veno-venous-ECMO’:ti,ab,kw,de OR ‘VA-ECMO’:ti,ab,kw,de OR ‘VV-ECMO’:ti,ab,kw,de OR ‘venoarterial ECMO’:ti,ab,kw,de OR ‘venovenous ECMO’:ti,ab,kw,de OR ‘veno-arterial extracorporeal membrane oxygenation’:ti,ab,kw,de OR ‘veno-venous extracorporeal membrane oxygenation’:ti,ab,kw,de OR ‘venoarterial extracorporeal membrane oxygenation’:ti,ab,kw,de OR ‘venovenous extracorporeal membrane oxygenation’:ti,ab,kw,de)

Term concept #2: (‘heparin’/exp OR ‘thrombin inhibitor’/exp OR ‘anticoagulant agent’/exp OR ‘hirudin’/exp OR ‘bivalirudin’/exp OR ‘nafamstat mesilate’/exp OR ‘Bivalirudin*’:ti,ab,kw,de OR ‘Argatroban’:ti,ab,kw,de OR ‘Lepirudin’:ti,ab,kw,de OR ‘Nafamostat’:ti,ab,kw,de OR ‘Nafamostat mes?late’:ti,ab,kw,de OR ‘anticoagulation’:ti,ab,kw,de OR ‘anticoagulant’:ti,ab,kw,de OR ‘Direct thrombin inhibitor*’:ti,ab,kw,de OR ‘DTI’:ti,ab,kw,de OR ‘Heparin’:ti,ab,kw,de)

Term concept #3: (‘cerebrovascular accident’/exp OR ‘thromboembolism’/exp OR ‘bleeding’/exp OR ‘treatment outcome’/exp OR ‘mortality’/exp OR ‘survival’/exp OR ‘adverse event’/exp OR ‘adverse drug event’:ti,ab,kw,de OR ‘thromboembol*’:ti,ab,kw,de OR ‘complication’:ti,ab,kw,de OR ‘adverse events’:ti,ab,kw,de OR ‘bleeding’:ti,ab,kw,de OR ‘bleeding events’:ti,ab,kw,de OR ‘bleeding complications’:ti,ab,kw,de OR ‘hemorrhage’:ti,ab,kw,de OR ‘death’:ti,ab,kw,de OR ‘mortality’:ti,ab,kw,de OR ‘survival’:ti,ab,kw,de)

**Search for CINAHL:**

Term Concept #1: (MH "Extracorporeal Membrane Oxygenation") OR “Extracorporeal membrane oxygenation” OR “extracorporeal life support” OR ECMO OR ECLS OR “veno-arterial ECMO” OR “veno-venous-ECMO” OR “VA-ECMO” OR “VV-ECMO” OR “venoarterial ECMO” OR “venovenous ECMO” OR “veno-arterial extracorporeal membrane oxygenation” OR “veno-venous extracorporeal membrane oxygenation” OR “venoarterial extracorporeal membrane oxygenation” OR “venovenous extracorporeal membrane oxygenation”

Term concept #2: (MH "Heparin+") OR (MH "Anticoagulants+") OR “Bivalirudin*” OR Argatroban OR Lepirudin OR Nafamostat OR “Nafamostat mes?late” OR anticoagulation OR anticoagulant OR “Direct thrombin inhibitor*” OR DTI OR Heparin

Term concept #3: (MH "Adverse Health Care Event+") OR (MH "Embolism and Thrombosis+") OR (MH "Hemorrhage+") OR (MH "Treatment Outcomes+") OR (MH "Mortality+") OR (MH "Survival") OR “adverse drug event” OR “thromboembol*” OR complication OR “adverse events” OR bleeding OR “bleeding events” OR “bleeding complications” OR hemorrhage OR death OR mortality OR survival

**Cochrane:**

#1 MeSH descriptor: [Extracorporeal Membrane Oxygenation] explode all trees

#2 "Extracorporeal membrane oxygenation"

#3 "extracorporeal life support"

#4 "ECLS"

#5 "ECMO"

#6 "veno-arterial ECMO"

#7 "veno-venous ECMO"

#8 “VA-ECMO”

#9 “VV-ECMO”

#10 “venoarterial ECMO”

#11 "venovenous ECMO"

#12 "veno-arterial extracorporeal membrane oxygenation"

#13 "veno-venous extracorporeal membrane oxygenation"

#14 “venoarterial extracorporeal membrane oxygenation”

#15 “venovenous extracorporeal membrane oxygenation”

#16 #1 OR #2 OR #3 OR #4 OR #5 OR #6 OR #7 OR #8 OR #9 OR #10 OR #11 OR #12 OR #13 OR #14 OR #15

#17 MeSH descriptor: [Heparin] explode all trees

#18 "heparin"

#19 "unfractionated heparin"

#20 "low-molecular-weight heparin"

#21 MeSH descriptor: [Heparin, Low-Molecular-Weight] explode all trees

#22 MeSH descriptor: [Antithrombins] explode all trees

#23 "antithrombins"

#24 "direct antithrombins"

#25 "direct thrombin inhibitors"

#26 "dti"

#27 MeSH descriptor: [Hirudins] explode all trees

#28 "hirudin"

#29 "argatroban"

#30 "bivalirudin"

#31 "bivalirudin*"

#32 "lepirudin"

#33 "desirudin"

#34 "inogatran"

#35 "melagatran"

#36 "dabigatran"

#37 "ximelagatran"

#38 "nafamostat"

#39 "nafamostat mesylate

#40 MeSH descriptor: [Anticoagulants] explode all trees

#41 "anticoagulation"

#42 "anticoagulant"

#43 #17 OR #18 OR #19 OR #20 OR #21 OR #22 OR #23 OR #24 OR #25 OR #26 OR #27 OR #28 OR #29 OR #30 OR #31 OR #32 OR #33 OR #34 OR #35 OR #36 OR #37 OR #38 OR #39 OR #40 OR #41 OR #42

#44 MeSH descriptor: [Blood Coagulation] explode all trees

#45 "blood coagulation"

#46 "coagulopathy"

#47 MeSH descriptor: [Thrombosis] explode all trees

#48 "thrombosis"

#49 MeSH descriptor: [Embolism and Thrombosis] explode all trees

#50 "embolism and thrombosis"

#51 "thromboembolism"

#52 "thromboemb*"

#53 "thromboembolic events"

#54 "thromboembolic event"

#55 "thrombo-embolism"

#56 "complication"

#57 MeSH descriptor: [Hemorrhage] explode all trees

#58 "hemorrhage"

#59 "bleeding"

#60 "bleeding event"

#61 "bleeding complication"

#62 MeSH descriptor: [Mortality] explode all trees

#63 "mortality" 106744

#64 MeSH descriptor: [Death] explode all trees

#65 "death"

#66 "dying"

#67 MeSH descriptor: [Survival] explode all trees

#68 "survival"

#69 "survive"

#70 MeSH descriptor: [Drug-Related Side Effects and Adverse Reactions] explode all trees

#71 "drug-related side effects and adverse reactions"

#72 "drug-related side effects"

#73 "side effects"

#74 "adverse reactions"

#75 "drug-related adverse reactions"

#76 "adverse drug event"

#77 "adverse events"

#78 #44 OR #45 OR #46 OR #47 OR #48 OR #49 OR #50 OR #51 OR #52 OR #53 OR #54 OR #55 OR #56 OR #57 OR #58 OR #59 OR #60 OR #61 OR #62 OR #63 OR #64 OR #65 OR #66 OR #67 OR #68 OR #69 OR #70 OR #71 OR #72 OR #73 OR #74 OR #75 OR #76 OR #77

#79 #16 AND #43 AND #78
