# Supplementary material for: Evaluation of clinical outcomes in patients treated with heparin or direct thrombin inhibitors during extracorporeal membrane oxygenation: a systematic review and meta-analysis
Source: Thromb J. 2022 Jul 28;20:42. doi: 10.1186/s12959-022-00401-2 (PMC9330661; doi:10.1186/s12959-022-00401-2)
Supplement: Supplementary file 4 — Additional file 4: Table S1. Detailedcharacteristics of included studies. [file 12959_2022_401_MOESM4_ESM.docx]

**Table S1: Detailed characteristics of included studies**

| Author | Year of publication | title | Study design | Type of Comparison | Number of participants per group | Adult / pediatric patients | Type of ECMO | Indication for ECMO | Male sex | Mean age ± SD (years) | aPTT aim (s) | Percentage of time within therapeutic aPTT | Time to aPTT goal (h) | Length of ECMO therapy | Length of hospital stay | In-hospital mortality (# of patients) | Cum. Thrombotic events (# of patients) | Patient related thrombosis (# of patients) | Device related thrombosis (# of patients) | Cum. Bleeding (# of patients) | Major bleeding (# of patients) | Minor bleeding (# of patients) |
| --- | --- | --- | --- | --- | --- | --- | --- | --- | --- | --- | --- | --- | --- | --- | --- | --- | --- | --- | --- | --- | --- | --- |
| Hamzah | 2022  (under review) | Bivalirudin versus Heparin for Systemic Anticoagulation during Pediatric Extracorporeal Membrane Oxygenation: A Contemporary Multicenter Comparative Analysis | Multi center, retrospective | Heparin vs. Bivalirudin | Total: 225  Hep.: 150  Biv.: 75 | pediatric | VV-ECMO: 36  VA-ECMO: 141  eCPR: 48 | eCPR: 48  CPB weaning: 115  Not reported: 62 | Hep.: 74  Biv.: 38 | Hep.: 8 (1, 36)  Biv.: 7 (2, 37)  (months, median, IQR) | Not reported | Hep.: 47.5±31  Biv.: 72.6±19.1 | Hep.: 16.3±14.63  Biv.: 11.9±9.6 | Hep.: 155,4±160,2  Biv.: 183,1±257 (h) | Not reported | Hep.: 69  Biv.: 23 | Hep.: 42  Biv.: 12 | Hep.: 42  Biv.: 12 | Not reported | Hep.: 129  Biv.: 45 | Hep.: 129  Biv.: 45 | Not reported |
| Pieri | 2021 | "Eleven Years of Venovenous Extracorporeal  Membrane Oxygenation for Acute Respiratory Distress  Syndrome: From H1N1 to SARS-CoV-2. Experience  and Perspectives of a National Referral Center" | Single center, retrospective | Heparin vs. Bivalirudin | Total: 125  Hep.: 26  Biv.: 99 | All adult patients | VV-ECMO only | ARDS only | Total: 93 | Not reported | Hep.: 55-60  Biv.:55-60 | Not reported | Not reported | Hep.: 10±8  Biv.: 16±16 (d) | Not reported | Hep.: 20  Biv.: 50 | Hep.: 3  Biv.: 10 | Hep.: 3  Biv.: 10  (peripheral ischemia, stroke) | Not reported | Not reported | Hep.: 7  Biv.: 21 | Hep.: 15  Biv.: 37 |
| Sheridan | 2021 | "Comparison of Bivalirudin Versus Unfractionated Heparin  for Anticoagulation in Adult Patients on Extracorporeal  Membrane Oxygenation" | Single center, retrospective | Heparin vs. Bivalirudin | Total: 150  Hep.: 50  Biv.: 100 | All adult patients | VV-ECMO: 52  VA-ECMO: 88 | CS: 58  Resp. fail.: 59  PE: 10  CPB weaning: 11  Others:12 | Total: 106  Hep.: 32  Biv.: 74 | Total: 53±14.5  Hep.: 53±14  Biv.: 54±15 | Hep.: <95 and anti-FXa 0.3–0.7 IU/mL  Biv.:45-75 | Hep.: 22 (6, 47.3)  Biv.: 86 (76, 100)  (median, IQR) | Hep.: 18 (12, 34.5)  Biv.: 2 (2, 6)  (median, IQR) | Hep.: 6 (4, 10)  Biv.: 6 (4, 9) (d)  (median, IQR) | Hep.: 19 (11, 33)  Biv.: 16 (8, 26) (d)  (median, IQR) | Hep.: 25  Biv.: 57 | Not reported | Hep.: 0  Biv.: 1 | Hep.: 2  Biv.: 4  (circuit failure) | Not reported | Hep.: 2  Biv.: 6 | Hep.: 4  Biv.: 4 |
| Machado | 2021 | Bivalirudin May Reduce the Need for Red Blood Cell Transfusion in Pediatric Cardiac Patients on Extracorporeal Membrane Oxygenation | Single center, retrospective | Heparin vs. Bivalirudin | Total: 32  Hep.: 14  Biv.: 18 | All pediatric patients | VA-ECMO: 30  VV-ECMO: 1  Hybrid: 1 | Not reported | Total: 12  Hep.: 9  Biv.: 7 | Hep.: 39.8±76.1  Biv.: 36± 58.8 (months) | Not reported (individual goals) | Hep.: 57±11  Biv.: 54±14 | Hep.: 12.54±9.96  Biv.: 21.06±12.53 | Hep.: 154.8±107.7  Biv.: 166.6±65.1(h) | Not reported | Hep.: 6  Biv.: 6 | Not reported | Hep.: 4  Biv.: 1  (LV-clot, limb ischemia) | Hep.: 5  Biv.: 4  (circuit failure) | Hep.: 5  Biv.: 2 | Hep.: 5  Biv.: 2 | Not reported |
| Seelhammer | 2021 | Comparison of Bivalirudin Versus Heparin for Maintenance Systemic Anticoagulation During Adult and Pediatric Extracorporeal Membrane Oxygenation | Single center, retrospective | Heparin vs. Bivalirudin | Total: 422  Hep.: 288  Biv.: 134 | Adult: 333  Pediatric: 89 | VA-ECMO: 358  VV-ECMO: 64 | Post cardiotomy: 162  CS: 100  Resp. fail.: 86  eCPR: 69  Transplant: 5 | Total: 265  Hep.: 183  Biv.: 82 | Not reported | Hep.: 60-90  Biv.: 60-80 | Not reported | Not reported | Hep.: 5,1 (2.9, 10.2) [adult]; 7,2 (4.2, 18.6) [pediatric]  Biv.: 4,7 (2.8, 8.8) [adult]; 5,3 (2.7, 13.8) [pediatric] (d)  (median, IQR) | Hep.: 17.7  (9.3, 37.5) [adult]; 35.8  (16.0, 72.9) [pediatric]  Biv.: 21.5 (10.1, 47.0) [adult]; 23.3 (12.8, 54.9) [pediatric] (d)  (median, IQR) | Hep.: 155 (total), 118 (adult), 37 (pediatric)  Biv.: 52 (total), 42 (adult), 10 (pediatric) | Not reported | Hep: 52 (total), 38 (adult), 14 (pediatric)  Biv.: 16 (total), 13 (adult), 3 (pediatric) | Hep: 74 (total), 44 (adult), 30 (pediatric)  Biv.: 14 (total), 6 (adult), 8 (pediatric)  (circuit interventions) | Not reported | Not reported | Not reported |
| Schill | 2021 | Is anticoagulation with bivalirudin comparable to heparin for pediatric extracorporeal life support? Results from a high-volume center | Single center, retrospective | Heparin vs. Bivalirudin  vs. switched | Total: 54  Hep.: 34  Biv.: 14  Switched: 8 | All pediatric patients | VA-ECMO: 38  VV-ECMO: 18 | Post cardiotomy: 20  Resp. fail.: 19  CS: 17 | Not reported | Hep.: 16.3 (4.8, 143.7)  Biv.: 5.5 (3.7, 79.6) (months, median,IQR) | Hep.: anti-FXa 0.3–0.7 IU/mL  Biv.: 60-95 | Not reported | Not reported | Hep.: 3.3 (2.1, 6.2)  Biv.: 11.0 (6.2, 23.1) (d)  (median, IQR) | Not reported | Hep.: 12  Biv.: 6 | Not reported | Hep.: 7  Biv.: 1  (ischemic stroke) | Not reported | Not reported | Hep.: 4  Biv.: 1  (hemorrhagic stroke) | Not reported |
| Kaushik | 2021 | Use of bivalirudin for anticoagulation in pediatric extracorporeal membrane oxygenation (ECMO) | Single center, retrospective | Heparin vs. Bivalirudin vs. switched | Total: 39  Hep.: 27  Biv.: 8  Switched: 4 | All pediatric patients | VA-ECMO:34  VV-ECMO: 4  Hybrid: 1 | Resp. Fail.: 12  CS: 11  eCPR: 6 | Total: 20  Hep.: 15  Biv.: 3 | Hep.: 4.0 (0.5, 92.0)  Biv.: 0.6 (0.0, 80.0) (months, median, IQR) | Hep.: 60-90  Biv.: 60-90 | Hep.: 44.0 (21.0, 53.0)  Biv.: 65.0 (47.5, 72.0)  (median, IQR) | Hep.: 12 (5.75,26)  Biv.: 14.5 (6.7,16)  (median, IQR) | Hep.: 6.0 (3.0, 9.0)  Biv.: 12.0 (5.5, 23.5) (d)  (median, IQR) | Hep.: 33.5 (14.0, 91.0)  Biv.: 26.0 (14.5, 60.5) (d)  (median, IQR) | Hep.: 7  Biv.: 5  (death during ECMO) | Not reported | Not reported | Hep.: 3  Biv.: 2  (circuit change) | Not reported | Hep.: 12  Biv.: 1 | Hep.: 1  Biv.: 1 |
| Rivosecchi | 2021 | Comparison of Anticoagulation Strategies in Patients Requiring Venovenous Extracorporeal Membrane Oxygenation: Heparin Versus Bivalirudin | Single center, retrospective | Heparin vs. Bivalirudin | Total: 295  Hep.: 162  Biv.: 133 | All adult patients | VV-ECMO only | Resp. fail.: 145  Pre/post-transplant: 108  Post cardiotomy: 20  Others: 22 | Total: 146  Hep.: 95  Biv.: 81 | Hep.: 49 (36,61)  Biv.: 49 (36,61) (median, IQR) | Hep.: anti-FXa 0.25–0.35 IU/mL  Biv.: 60-75 | Not reported | Not reported | Hep.: 238.8±333.0Biv.: 229.2±284.7 (h) | Hep.: 44.4±47.3  Biv.: 35.3±31.1(d) | Hep.: 74  Biv.: 46  (1-year mortality) | Not reported | No difference reported (p-value 0.43 no further information) | Hep.: 53  Biv.: 23  (circuit thrombosis) | Not reported | Hep.: 66  Biv.: 16 | Not reported |
| Fisser | 2021 | Argatroban versus heparin in patients without heparin-induced thrombocytopenia during venovenous extracorporeal membrane oxygenation: a propensity-score matched study | Single center, retrospective | Heparin vs. Argatroban | Total: 117  Hep.: 78  Arg.: 39 | All adult patients | VV-ECMO only | ARDS only | Total: 80  Hep.: 51  Arg.: 29 | Hep.: 56 (48,63)  Arg.: 55 (46,61) (median, IQR) | Hep.: 45-55  Arg.:45-55 | Not reported | Not reported | Hep.: 13.56±11.96  Arg.: 14.74±13.29 (d) | Hep.: 32.3±31.4  Arg.: 32.4±28.9(d) | Hep.:31  Arg.:9 | Not reported | Hep.: 32  Arg.: 22  (major and minor deep vein thrombosis) | Hep.: 5  Arg.: 1  (circuit exchange) | Hep.: 71  Arg.: 34 | Hep.: 63  Arg.: 27 | Hep.: 8  Arg.: 7 |
| Cho | 2021 | Cost-effectiveness of Argatroban Versus Heparin Anticoagulation in Adult Extracorporeal Membrane Oxygenation Patients | Single center, retrospective | Heparin vs. Argatroban | Total: 35  Hep.: 24  Arg.: 11 | All adult patients | VA-ECMO: 10  VV-ECMO: 21  Hybrid: 4 | Not reported | Total: 22  Hep.: 15  Arg.: 7 | Total: 46±17  Hep.: 45±16  Arg.: 49±20 | Hep.: 40-60 or 60-80 (high dose)  Arg.:43-85 | Hep.: 54  Arg: 62  (no SD reported) | Hep.: 7±9  Arg.: 5±4 | Hep.: 180±153  Arg.: 136±57 (h) | Hep.: 23±13  Arg.: 26±11 days (d) | Hep.:5  Arg.:1 | Hep.: 15  Arg.: 6 | Hep.: 2  Arg.: 0  (deep vein thrombosis, ischemic events) | Hep.: 15  Arg.: 6  (in circuit clotting) | Hep.: 15  Arg.: 5 | Hep.: 2  Arg.: 1 | Hep.: 13  Arg.: 4 |
| Hamzah | 2020 | Evaluation of Bivalirudin As an Alternative to Heparin for Systemic Anticoagulation in Pediatric Extracorporeal Membrane Oxygenation | Single center, retrospective | Heparin vs. Bivalirudin | Total: 32  Hep.: 16  Biv.: 16 | All pediatric patients | VA-ECMO: 29  VV-ECMO: 3 | Post cardiotomy:13  Others: not reported | Total: 14  Hep.: 8  Biv.: 6 | Total: 12 (0–212)  Hep.: 59 (0, 212)  Biv.: 31 (0–99) (months, median, IQR) | Hep.: 60-80  Biv.: 58-78 or 50-70 (open chest) | Not reported | Hep.: 38.67±31.62  Biv.: 16.5±15.98 | Hep.: 136.3±96.4  Biv.: 137.7±87.01 (h) | Not reported | Hep.: 5  Biv.: 3 | Hep.: 3  Biv.: 0 | Hep.: 2  Biv.: 0  (stroke) | Hep.: 1  Biv.: 0  (circuit thrombosis) | Hep.: 12  Biv.: 3 | Hep.: 12  Biv.: 3 | Not reported |
| Kaseer | 2020 | Heparin vs bivalirudin anticoagulation for extracorporeal membrane oxygenation | Single center, retrospective | Heparin vs. Bivalirudin | Total: 52  Hep.: 33  Biv.: 19 | All adult patients | VA-ECMO: 28  VV-ECMO: 24 | CS:15  ARDS:24  Transplant: 17  Others:1 | Total: 37  Hep.: 25  Biv.: 12 | Total: 55 (18, 83)  Hep.: 53 (21, 83)  Biv.: 56 (18, 71) (median, IQR) | Hep.: 50-70 or 40-60  Biv.: 60-90 or 50-70 | Hep.: 44±30  Biv.: 69±36 | Not reported | Hep.: 258±173  Biv.: 443±404 (h) | Not reported | Hep.: 15  Biv.: 7 | Hep.: 11  Biv.: 5 | Hep.: 3  Biv.: 1  (Arterial thromboembolism) | Hep.: 9  Biv.: 5  (in circuit thrombosis) | Hep.: 6  Biv.: 1 | Hep.: 6  Biv.: 1 | Not reported |
| Macielak | 2019 | Impact of anticoagulation strategy and agents on extracorporeal membrane oxygenation therapy | Single center, retrospective | Heparin vs. Bivalirudin vs. switched | Total: 153  Hep.: 100  Biv.: 10  Switched: 43 | All adult patients | VA-ECMO: 134  Other types not reported | Salvage: 61%  CS: 46%  ARDS:29%  Resp. fail.: 29%  CPB weaning: 23%  Others: 12% | Total: 127 | Total: 52.8±14.2  Hep.: 51.4±14.0  Biv.: 57.9±13.8 | Hep.: 72-95  Biv.: 60-80 | Hep.: 37.72±18.98  Biv.: 52.72±10.66 | Not reported | Hep.: 7.1±4.8  Biv.: 6.4±2.8 (d) | Hep.: 24.5 (16.5, 44.5)  Biv.: 17.5 (15, 36) (d)  (median, IQR) | Total: 54  (not reported for anticoagulants) | Not reported | Not reported | Not reported | Not reported | Not reported | Not reported |
| Berei | 2018 | Evaluation of Systemic Heparin Versus Bivalirudin in Adult Patients Supported by Extracorporeal Membrane Oxygenation | Single center, retrospective | Heparin vs. Bivalirudin | Total: 72  Hep.: 28  Biv.: 44 | All adult patients | VA-ECMO: 66  VV-ECMO: 6 | CS: 51  Sepsis: 11  Resp. fail.: 4  Others: 6 | Total: 47  Hep.: 18  Biv.: 29 | Hep.: 55.9±13.1  Biv.: 55.2±15.2 | Hep.: 45-65 or 65-90  Biv.: 45-65 or 65-90 | Hep.: 83.02±21.90  Biv.: 87,71±16.30 | Hep.: 14.88 (8.81, 22.80)  Biv.: 4.50 (3.38, 8.00) (median, IQR) | Hep.: 162.1±125.5  Biv.: 156.9±112.3 (h) | Hep.: 22.5 (11.8, 36.5)  Biv.: 16.5 (12.0, 39.3) (d)  (median, IQR) | Hep.: 9  Biv.: 16 | Hep.: 7  Biv.: 10 | Hep.: 6  Biv.: 8 | Hep.: 1  Biv.: 1  (pump thrombosis) | Not reported | Hep.: 7  Biv.: 20 | Hep.: 7  Biv.: 10 |
| Menk | 2017 | Efficacy and safety of argatroban in patients with acute respiratory distress syndrome and extracorporeal lung support | Single center, retrospective | Heparin vs. Argatroban | Total: 78  Hep.: 39  Arg.: 39 | All adult patients | VV-ECMO: 43  pECLA: 24  Hybrid: 11 | ARDS only | Total: 54  Hep.: 27  Arg.: 27 | Hep.: 48 (35,64)  Arg.: 47 (36,60) (median, IQR) | Hep.: 50-75  Arg.:50-75 | Not reported | Not reported | Hep.: 428 (180,652)  Arg.: 265 (131,460) (h)  (median, IQR) | Not reported | Hep.: 22  Arg.: 21  (ICU mortality) | Not reported | Hep.: 3  Arg.: 6 | Hep.: 11  Arg.: 15  (ECMO Oxygenator change) | Not reported | Hep.: 13  Arg.: 11 (whole cohort) | Hep.: 31  Arg.: 30 (whole cohort) |
| Ljajikj | 2017 | Bivalirudin anticoagulation for left ventricular assist device implantation on an extracorporeal life support system in patients with heparin-induced thrombocytopenia antibodies | Single center, retrospective | Heparin vs. Bivalirudin | Total: 20  Hep.: 10  Biv.: 10  (after PS-Matching) | All adult patients | VA-ECMO only | support pre, during and after LVAD implantation only | Total: 17  Hep.: 9  Biv.: 8 | Hep.: 52.5±9.7  Biv.: 48.2±14.1 | Not reported | Not reported | Not reported | Not reported | Not reported | Hep.: 6  Biv.: 5  (1-year mortality) | Not reported | Hep.: 2  Biv.: 1  (stroke) | Not reported | Not reported | Hep.: 1  Biv.: 3  (chest washouts and hemorrhagic stroke) | Not reported |
| Pieri | 2013 | Bivalirudin versus heparin as an anticoagulant during extracorporeal membrane oxygenation: a case-control study | Single center, retrospective | Heparin vs. Bivalirudin | Total: 20  Hep.: 10  Biv.: 10 | All adult patients | VA-ECMO: 10  VV-ECMO: 10 | Not reported | Total: 16  Hep.: 9  Biv.: 7 | Hep.: 54±12.7  Biv.: 59.5±14.4 | Hep.: 45-60  Biv.: 45-60 | Hep.: 174/200 (74%)  Biv.: 192/216 (88.9%)  (no SD reported) | Not reported | Hep.: 5.7±5.49  Biv.: 11±12 (d) | Hep.: 10±4.7  Biv.: 27±14  (d) | Hep.: 5  Biv.: 4 | Hep.: 3  Biv.: 1 | Hep.: 2  Biv.: 1 | Hep.: 1  Biv.: 0 | Not reported | Hep.: 4  Biv.: 3 | Hep.: 2  Biv.: 0 |
| Ranucci | 2011 | Bivalirudin-based versus conventional heparin anticoagulation for postcardiotomy extracorporeal membrane oxygenation | Single center, retrospective | Heparin vs. Bivalirudin | Total: 21  Hep.: 8  Biv.: 13 | Adult: 12  Pediatric: 9 | VVA-ECMO: 21 | Post cardiotomy only | Not reported | Hep.: 13.9±19  Biv.: 36.5±29 | Hep.: 50-80  Biv.: 50-80 | Not reported | Not reported | Hep.: 80±52  Biv.: 143±73 (h) | Not reported | Hep.: 6  Biv.: 10 | Not reported | Hep.: 1  Biv.: 0  (stroke) | Hep.: 1  Biv.: 1  (pump head thrombisis) | Not reported | Not reported | Not reported |

**Legend:** The table shows detailed characteristics of studies included into analysis. For all outcomes number of patients are reported separately for heparin (hep.), bivalirudin (biv.) and argatroban (arg.) if applicable. Continuous outcomes are reported as mean with standard deviation (SD) or median with interquartile range (IQR), as reported by the authors. (abbreviations: aPTT=activated partial thromboplastin time, ARDS = acute respiratory distress syndrome, CPB = cardiopulmonary bypass, CS = cardiogenic shock, cum.=cumulative, ECMO=extracorporeal membrane oxygenation, eCPR= extracorporeal cardiopulmonary resuscitation, LVAD = left ventricular assist device, PE = pulmonary embolism, Resp. fail = respiratory failure, VA=Venoarterial, VV=venovenous)
