# Supplementary material for: Evaluation of clinical outcomes in patients treated with heparin or direct thrombin inhibitors during extracorporeal membrane oxygenation: a systematic review and meta-analysis
Source: Thromb J. 2022 Jul 28;20:42. doi: 10.1186/s12959-022-00401-2 (PMC9330661; doi:10.1186/s12959-022-00401-2)
Supplement: Supplementary file 5 — Additional file 5: Table S2. Definitions of outcomes. [file 12959_2022_401_MOESM5_ESM.docx]

**Table S2: Definitions of outcomes**

| **Author** | **Year of publication** | **Major bleeding** | **Minor bleeding** | **Pump related thrombosis** | **Patient related thrombosis** |
| --- | --- | --- | --- | --- | --- |
| Hamzah | 2022  (under review) | decrease of hemoglobin by 2 g/dl, transfusion of PRBCs >20 mL/kg over 24 hours. Central nervous system bleeding or bleeding requiring surgical intervention. | Not reported | exchange of any component of the circuit due to thrombosis | thromboembolic events to the brain, visceral organs, or extremities. |
| Pieri | 2021 | decrease of hemoglobin by 2 g/dl, transfusion of PRBCs >10 mL/kg over 24 hours. Central nervous system, retroperitoneal or pulmonary bleeding or bleeding requiring surgical intervention. | Not defined | Not reported | Peripheral ischemia and ischemic stroke |
| Sheridan | 2021 | Bleeding academic research consortium (BARC) criteria III | Bleeding academic research consortium (BARC) criteria I-II | Circuit failure requiring exchange | New venous or arterial thromboembolism stroke or intracardiac thrombus. |
| Machado | 2021 | Bleeding requiring surgical intervention, end-organ hemorrhage, fatal bleeding | Not reported | Thrombosis that leads to circuit exchange | Left ventricular clot or limb ischemia |
| Seelhammer | 2021 | Not defined | Not defined | Thrombosis leading to circuit interventions | Stroke, seep vein thrombosis, pulmonary embolism myocardial infarction or mesenteric ischemia |
| Schill | 2021 | Hemorrhagic stroke | Not reported | Not reported | Ischemic stroke |
| Kaushik | 2021 | Surgical mediastinal or cannula site intervention. Bleeding requiring VIIa administration, gastrointestinal, pulmonary or intracranial hemorrhage or bleeding requiring transfusion. | Not defined | Thrombosis that leads to circuit exchange | Not reported |
| Rivosecchi | 2021 | Major bleeding according to ELSO criteria | Minor bleeding according to ELSO criteria | In circuit thrombosis with visible thrombosis on any portion of ECMO requiring change in Cannula, pump or oxygenator | Non-circuit related thrombotic complications retrieved from clinical progress notes, imaging, or diagnostic tests documented in the medical records. |
| Fisser | 2021 | Major bleeding according to ELSO criteria (drop of hemoglobin >2g/dl/d, transfusion >2 PRBC/24h, retroperitoneal, cerebral or pulmonary bleeding) | Transfusion of <2 PRBC/24h | Thrombosis that leads to circuit exchange | Major (>50% obstruction of the vessel) and minor (<50% obstruction of the vessel) deep vein thrombosis in area of cannulation (incompressibility of the vein and absence or reduction of flow) |
| Cho | 2021 | Not defined | Not defined | In circuit clotting | Deep vein thrombosis and ischemic events |
| Hamzah | 2020 | decrease of hemoglobin by 2 g/dl, transfusion of PRBCs >20 mL/kg over 24 hours. Central nervous system bleeding or bleeding requiring surgical intervention. | Not reported | Thrombosis that leads to circuit exchange | Thromboembolic events to brain, visceral organs or extremities. |
| Kaseer | 2020 | decrease of hemoglobin by 3 g/dl within 24h, inappropriate response to transfusions. | Not reported | ECMO in-circuit thrombosis | Arterial or venous thromboembolism |
| Macielak | 2019 | Major bleeding according to ELSO criteria (drop of hemoglobin >2g/dl/d, transfusion 1 or more 10/ml/kg PRBC/24h, retroperitoneal, cerebral or pulmonary bleeding, bleeding requiring surgical intervention cannula site bleeding) | Not reported | Requirement of oxygenator or circuit exchange | Venous thromboembolism, intracardiac thrombus, ischemic stroke |
| Berei | 2018 | Drop in hemoglobin of >3mg/dL within 24h. | Drop in hemoglobin of <3mg/dL within 24h. | Thrombus within ECMO circuit | Venous or arterial thromboembolism |
| Menk | 2017 | Intracranial, pulmonary, retroperitoneal or gastrointestinal bleeding found clinically or with imaging techniques | Bleeding from site of puncture for central venous lines, the nasopharyngeal zone or skin lesions | Clotting of ECMO oxygenator | Deep vein thrombosis, pulmonary embolism, limb ischemia, ischemic stroke |
| Ljajikj | 2017 | Chest washout and hemorrhagic stroke | Not reported | Not reported | Ischemic stroke |
| Pieri | 2013 | Not defined | Overt bleeding not meeting criteria for major bleeding | ECMO oxygenator related thrombosis | Venous or arterial occlusion with clinical signs and symptoms or evident at the radiologic examination |
| Ranucci | 2011 | Not reported | Not reported | Pump head thrombosis | Ischemic stroke |

**Legend:** The table shows the definitions for major and minor bleeding as well as for pump and patient related thrombosis for each individual included study. (Not reported = study does not report for this outcome; Not defined = study did not provide definition for this outcome)
